# Epilepsy, Behavioral Abnormalities, and Physiological Comorbidities in Syntaxin-Binding Protein 1 (STXBP1) Mutant Zebrafish

**DOI:** 10.1371/journal.pone.0151148

**Published:** 2016-03-10

**Authors:** Brian P. Grone, Maria Marchese, Kyla R. Hamling, Maneesh G. Kumar, Christopher S. Krasniak, Federico Sicca, Filippo M. Santorelli, Manisha Patel, Scott C. Baraban

**Affiliations:** 1 Department of Neurological Surgery, University of California San Francisco, San Francisco, California, United States of America; 2 Molecular Medicine & Clinical Neurophysiology Laboratories, IRCCS Stella Maris, Pisa, Italy; 3 Department of Pharmaceutical Sciences, University of Colorado Anschutz Medical Campus, Aurora, Colorado, United States of America; 4 Department of Biology, Colby College, Waterville, Maine, United States of America; National Institutes of Health / NICHD, UNITED STATES

## Abstract

Mutations in the synaptic machinery gene syntaxin-binding protein 1, *STXBP1* (also known as *MUNC18-1*), are linked to childhood epilepsies and other neurodevelopmental disorders. Zebrafish *STXBP1* homologs (*stxbp1a* and *stxbp1b*) have highly conserved sequence and are prominently expressed in the larval zebrafish brain. To understand the functions of *stxbp1a* and *stxbp1b*, we generated loss-of-function mutations using CRISPR/Cas9 gene editing and studied brain electrical activity, behavior, development, heart physiology, metabolism, and survival in larval zebrafish. Homozygous *stxbp1a* mutants exhibited a profound lack of movement, low electrical brain activity, low heart rate, decreased glucose and mitochondrial metabolism, and early fatality compared to controls. On the other hand, homozygous *stxbp1b* mutants had spontaneous electrographic seizures, and reduced locomotor activity response to a movement-inducing “dark-flash” visual stimulus, despite showing normal metabolism, heart rate, survival, and baseline locomotor activity. Our findings in these newly generated mutant lines of zebrafish suggest that zebrafish recapitulate clinical phenotypes associated with human syntaxin-binding protein 1 mutations.

## Introduction

Mutations in the human syntaxin-binding protein 1 (*STXBP1*) gene are associated with a range of clinical outcomes. *STXBP1* mutations were first identified in children with early infantile epileptic encephalopathy with burst suppression (EIEE; also known as Ohtahara Syndrome) [1, 2] and subsequently found in patients diagnosed with other forms of epileptic encephalopathy including infantile spasms [3, 4], Lennox-Gastaut Syndrome [4], and Dravet Syndrome [5]. These epilepsies are primarily pediatric, catastrophic, pharmacoresistant, and associated with intellectual disability. Additionally, *STXBP1* mutations are sometimes associated with non-syndromic intellectual disability without epilepsy [6], or with ataxia or dyskinesia [7] that can persist even though EEG paroxysmal abnormalities and seizures resolve spontaneously [8]. Intriguingly, impaired mitochondrial respiratory chain function was noted in some patients [9, 10]. Although studies of *STXBP1* function in animal models have revealed much of its biochemical mechanism, further insight is needed to understand how the full range of associated disease processes develop.

*STXBP1* homologs are evolutionarily conserved and play an essential role in vesicle release in *Drosophila melanogaster* [11], *Mus musculus* [12], and *Caenorhabditis elegans* [13]. STXBP1 protein is primarily found in the brain and interacts with syntaxins 1, 2, and 3 [14–16]. STXBP1 gates a conformational switch in the core SNARE machinery that facilitates docking of vesicles with the cell membrane [17, 18]. Homozygous *STXBP1* mutations in model organisms cause defects in synaptic vesicle docking and disrupt normal neural activity. Seizure phenotypes were not reported in heterozygous *Stxbp1*^*+/-*^, but these animals did show increased fear responses as measured by heart rate [19]. *STXBP1* mutations could also contribute to comorbidities associated with epileptic encephalopathies, which include disrupted sleep and metabolic circadian rhythms [20], neurodevelopmental delay [1–5], and decreased heart function [21]. Despite progress in understanding basic functions of *STXBP1*, exploring the consequences of *STXBP1* mutations would benefit from well-characterized animal models.

Zebrafish are a leading organism with which to model human neurological disease [22–24], and epilepsy in particular [25–32]. Within three to five days of fertilization, zebrafish develop from fertilized eggs into freely swimming larvae with a complex nervous system capable of sophisticated behaviors and susceptible to seizures. Mutant zebrafish lines from ethylnitrosourea (ENU) mutagenesis screens have allowed the study of genetic epilepsies, including Lowe’s syndrome (*ocrl1*) [33], and Dravet syndrome (*scn1lab*) [34]. To study the function of *STXBP1* in zebrafish, we used CRISPR/Cas9 gene editing to generate stable mutant lines for *stxbp1a* and *stxbp1b*, the zebrafish *STXBP1* homologues. In these mutants, we found spontaneous recurring seizures (i.e. epilepsy) and significant defects in development, locomotor activity, and metabolic rate. Overall, our results demonstrate that zebrafish *stxbp1a* and *stxbp1b* have conserved roles in epilepsy and metabolic, physiological, and behavioral development.

## Materials and Methods

### Zebrafish Maintenance

Adult male and female wild-type zebrafish (TL and WIK strains) were obtained from the Zebrafish International Resource Center (Eugene, OR). Zebrafish were maintained according to standard procedures [35] and following guidelines approved for this study by the University of California, San Francisco Institutional Animal Care and Use Committee, Approval Number AN108659-01D. The zebrafish room was maintained on a 14hr light:10hr dark cycle, with lights-on at 9:00AM and lights-off at 11:00PM. Fish system water conditions were maintained in the following ranges by automated feedback controls: 29–30°C, pH 7.5–8.0, conductivity (EC) 690–710. Zebrafish embryos and larvae were raised in an incubator maintained at 28.5°C, on the same light-dark cycle as the fish facility. Water used for embryos and larvae was made by adding 0.03% Instant Ocean and 0.000002% methylene blue to reverse-osmosis distilled water. Embryos and larvae were raised in plastic petri dishes (90mm diameter, 20mm depth) and their housing density was limited to approximately 60 individuals per dish.

### In Situ Hybridization

Templates for riboprobes were generated by PCR amplification of zebrafish *stxbp1a* (ENSDARG00000001994) and *stxbp1b* (ENSDARG00000056036) from zebrafish cDNA. Probes were designed to be complementary to a 690-bp region spanning from exon 7 to exon 14 for *stxbp1a*, and a 800-bp region spanning from exon 13 to 3’UTR in exon 20 for *stxbp1b*. The T3 promoter sequence (aattaaccctcactaaaggg) was added to either the reverse primer (for the antisense probe) or the forward primer (for the sense control). Primer Sequences: stxbp1a-Fprobe: CTGACGCCTTCCAGAGTTTC, stxbp1a-Rprobe: CCTCAGCATCTGTTCCCATT; stxbp1b-Fprobe; AGTACCAGGGCACAGTGGAC, stxbp1b-Rprobe; AACAGTGAGGTGGGGCTATG. Digoxigenin-labeled riboprobes for *stxbp1a* were transcribed in vitro using standard reagents including DIG RNA labeling mixes (Roche Molecular Biochemicals), Fermentas T3 RNA Polymerase, RNAse out, and were then treated with DNAse and purified using GE Healthcare illustra ProbeQuant G-50 Micro Columns.

ISH was performed following standard protocols. Larvae were fixed in 4% paraformaldehyde (pH 7.0) in phosphate-buffered saline (PBS) at 4°C overnight. Following fixation, larvae were rinsed 4X for 5 minutes in PBS with 0.1% Tween 20 (PBSTw). Larvae were then rinsed with 100% methanol for 5 minutes, transferred to fresh methanol, and subsequently stored in 100% methanol at -20°C until needed. Before experimentation, brains and larvae were rehydrated in PBS, and brains were dissected using forceps. Brains were then processed and stained in wells of a 24-well plate in incubation baskets (Intavis AG). Brains were rehydrated through a series of 5-minute washes in 75%, 50%, 25% methanol in PBS with 0.1% Tween (PBS/Tw). Brains were then washed twice for 5 minutes in PBS/Tw, treated with proteinase K for 5 to 10 minutes, and rinsed 4 times with PBS/Tw. Brains were fixed again with 4% PFA for 10 minutes at room temperature, washes 4 X5 min in PBST. Prehybridization (1 hour) and hybridization (overnight) were carried out at 58°C, in a hybridization buffer containing 50% Formamide, 5X SSC buffer, 5mg/mL yeast torula RNA, and 0.1% Tween. Posthybridization washes were carried out at 58°C: twice for 30 minutes in 50% Formamide, 2X SSC w/Tw, once for 15 minutes in 2X SSC w/Tw, and twice for 30 minutes in 0.2X SSC w/Tw. Tissues were then blocked for 1 hr in blocking buffer containing 100mM maleic acid, pH 7.2, 150mM NaCl, bovine serum albumen (BSA), sheep serum, and 0.1% Tween 20. Alkaline-phosphatase-conjugated anti-DIG Fab fragments (Roche) were then added to the blocking solution to a final dilution of 1:4000. Brains were incubated overnight at 4°C, then washed 4 times for at least 10 minutes each in Maleate Buffer and signal was revealed with NBT/BCIP (Roche), kept in the dark, at room temperature or 37°C. To stop the staining reaction, tissue was washed 5 times for 5 minutes in PBS w/Tw.

To identify the expression patterns of genes, embryos and 7 dpf larvae brains were visualized on a Nikon Eclipse Ni microscope and photographs taken with a color digital camera (Nikon DS-U3) controlled by Nikon NIS-Elements software (version 4.00.07). Stained sections were viewed in brightfield. For images of stained 7 dpf larvae brains, Nikon Elements software was used to create focused images from stacks of multiple images.

### Quantitative real-time PCR (qPCR)

Gene expression levels of *stxbp1a* and *stxbp1b* mRNA were examined using RNA pooled from 10 wild-type sibling larvae. Total RNA was extracted using Trizol^®^ Reagent (Invitrogen, Carlsbad, CA), treated with DNAse (Ambion/Applied Biosystems, Austin, TX) and quantified with NanoDrop^™^ ND-1000 spectrophotometer (Thermo Scientific). Reverse-transcription reactions were performed using SuperScript^™^III First-Strand Synthesis System (Invitrogen) with a mix of oligo(dT)_20_ and random hexamers. The cDNA templates were diluted 1:2 with DEPC sterile water. The qPCR reactions were performed using SybrGreen^®^ fluorescent master mix on an ABI Prism^®^ 7700 Sequence Detection System using ABI PRISM SDS v9.1 software (Applied Biosystems). Zebrafish *stxbp1a* cDNA was amplified using the following primers, which were designed using Primer Express v3.0 (Applied Biosystems): Stxbp1a_qF1: TTGCTGGATGCCAATGTCA; Stxbp1a_qR1: TCCGTGATCCCGTTCTTGAG. The following primers were used to amplify β-actin cDNA (GenBank Accession # FJ915059) for data normalization: β-actin-F: CATCCATCGTTCACAGGAAGTG; β-actin-R: TGGTCGTTCGTTTGAATCTCAT. Samples were run in triplicate and reactions contained 1× SYBR green master mix, 10 μM of each primer, and RNAse free water for a final volume of 10 μl. Samples without reverse transcriptase were run for each reaction as negative controls. Cycling parameters were as follows: 50°C × 2min, 95°C × 10min, then 40 cycles of the following 95°C × 15s, 60°C × 1min. For each sample a dissociation step was performed at 95°C × 15s, 60°C × 20s, and 95°C × 15s at the end of the amplification phase to check for the presence of primer dimers or non-specific products. For both genes, qPCR efficiencies were assessed by mean of 4-fold serial dilutions of pooled cDNA. Serially diluted cDNAs were used to construct standard curves and estimates of efficiencies, slope of the curves and the correlation coefficient. Triplicate quantification values (CT; cycle threshold) were analyzed using qCalculator software (programmed by Ralf Gilsbach) which estimates qPCR efficiency E = 10^(– 1/slope) and the relative gene expression between samples after normalization basing on both the Comparative ΔΔCT [36] and the Efficiency Based [37] methods.

### Establishing mutant lines

CRISPR/Cas9 mutations were generated in wild-type (TL strain) zebrafish using published techniques [38, 39]. Sequence-specific sgRNA template plasmids were generated for each target site by modifying DR274 (Addgene Plasmid #42250). Plasmid Dr274 was modified to contain gene-specific sequences selected using ZiFit software [40]. In order to avoid off-target genomic mutagenesis effects, which can occur at sites that closely resemble the target site, we selected target sites that have a minimum of three mismatches with every other site in the genome.

The sequences of the modified plasmids were verified by Sanger sequencing (Quintarabio). sgRNAs were transcribed from linearized template plasmids (Ambion MEGAscript T7/SP6), and purified (Ambion MegaClear Kit). Cas9 mRNA was transcribed in vitro from linearized template plasmid MLM3613 (Addgene Plasmid #42251).

Fertilized 1–2 cell stage zebrafish eggs were injected with an injection mix containing approximately 300ng/μl Cas9 mRNA and 15 ng/μl sgRNA. After injected eggs were incubated for one day, some were harvested to check for mutagenesis at the target site. DNA was extracted from pools of 10 injected embryos and uninjected controls, gDNA including the target site was amplified, and the target site was checked via Sanger sequencing. Multiple sequencing peaks were confirmed to be present at the sgRNA target site before proceeding.

Other Cas9/sgRNA-injected embryos were raised to adulthood. These F0-generation potential mutants were crossed, and DNA was extracted from pooled F1 embryos for PCR and sequencing, as performed on the injected embryos. To obtain stable lines with known mutations, F1 embryos were raised to adulthood and outcrossed to wild-type (WIK strain) zebrafish. For adult fish of each line, genomic DNA was extracted from tail tissue, amplified by PCR, cloned into TOPO pcr2.1 vector, and sequenced. Mutations were identified and multiple individuals from each line were sequenced to confirm that each individual carried the same mutation.

The newly generated mutant alleles are designated *stxbp1a*^s3000^ and *stxbp1b*^s3001^ using the University of California San Francisco (UCSF) “s” designation in accordance with the Zebrafish Information Network (ZFIN) guidelines.

### Genotyping

For genotyping, we extracted genomic DNA from whole larvae using the Zebrafish Quick Genotyping DNA Preparation Kit (Bioland Scientific). We amplified *stxbp1a* gDNA using the following primers: stxbp1a-F: CACACACTTACAGCAGGAATGAGTGG, stxbp1a-R: ATTCAGACTTCAACTGTACATGTATTGTG. These primers amplify a 275-bp region including the *stxbp1a* mutation site. The mutant allele was then detected by digesting the amplicon with BsaHI, for which the restriction site is absent in the mutant, and electrophoresis to separate the digested samples on a 1% agarose gel.

We amplified *stxbp1b* gDNA using the following primers stxbp1b-F: ATCTGCGTAGAAAGCTGAGCTTCATAG, stxbp1b-R: GTCAATGAAAATGGCACTAACTCCACACG. The mutant allele was then detected by digesting the amplicon with BsiHKAI, for which the restriction site is absent in the mutant, and electrophoresis to separate the digested samples on a 1% agarose gel.

### Morphological Phenotyping

Larvae were photographed using a SteREO Discovery.V8 microscope (Zeiss). Standard lengths (distance from the anterior tip of head to the base of the caudal fin) were measured manually using DanioScope software (Noldus, version 1.0.109).

### Behavioral Phenotyping

For locomotion tracking, single zebrafish larvae were placed in individual wells of a 96-well flat-bottomed Falcon culture dish (BD Biosciences). Each well contained approximately 200 μl of embryo medium. Behavior was monitored at room temperature (21–22°C) using a DanioVision system and EthoVision XT 8.0 locomotion tracking software (Noldus Information). Prior to conducting experiments, larvae still in their chorions at day 3 (homozygous mutants) had their chorions removed using forceps. 5 dpf larvae were allowed to acclimate to the tracking arena for 3 to 4 hours, and then 24 hours of continuous behavioral data were recorded beginning at 4:00PM. The light:dark cycle continued as usual: lights-off occurred at 11:00PM and lights-on at 9:00AM.

At 4:00PM, when 24 hour recordings completed, larvae responses to sudden changes in light intensity were tested. 6 dpf larvae were exposed to 10 seconds of darkness (0% intensity light) followed by 10 seconds of 100% intensity light.

Using EthoVision XT 8.0 software, distance, movement, and velocity parameters were analyzed for individual locomotion plots. JASP software was used for ANOVA tests (https://jasp-stats.org/).

### Heart Rate Measurements

Petri dishes containing 3 dpf larvae were removed from the incubator and allowed at least 10 minutes to acclimate to the light and temperature on the microscope stage. 30-second videos of individual unanesthetized fish were taken using 6.3x zoom magnification with a SteREO Discovery.V8 microscope (Zeiss) and an Axiocam ICm1 camera with ZEN 2012 software (Zeiss, version 1.1.2.0) at a frame rate of 26 fps. Videos were imported into DanioScope software (Noldus, version 1.0.109) as a compressed AVI file, where a subset of the beating heart was outlined and heart rate was calculated.

### Metabolic Measurements

Extracellular acidification rate and oxygen consumption rate, representing glycolysis and oxidative phosphorylation respectively, were measured simultaneously using an extracellular flux analyzer (XF24, Seahorse Biosciences). Prior to metabolic experiments, chorions were removed from homozygous mutant larvae using forceps. Single 5 dpf zebrafish larvae (n = 10 per group) were placed in individual wells of a 24-well islet plate and a fine screen mesh was placed to maintain the larvae in place. Rates were calculated as the mean of 3 measurements, each lasting 3 minutes, which were taken with 5 minutes between measurements.

### Survival

Larval zebrafish were maintained as described above and counted daily. After day 12 they were collected and genotyped. Data were plotted using Microsoft Excel.

### Electrophysiology

Electrophysiological recordings were carried out according to previously described methods [41, 42]. To obtain stable physiological recordings, zebrafish larvae were briefly anesthetized in tricaine, paralyzed in α-bungarotoxin and immobilized in 1.2% low-melting temperature agarose in zebrafish egg water. Larvae were embedded so that the dorsal aspect of the brain was accessible for electrode placement. Embedded larvae were bathed in egg water and visualized using an Olympus BX50 microscope (Olympus America Inc., Center Valley, PA). Under direct visual guidance, a glass microelectrode (b1.2 mm tip diameter, 2–7 MΩ) was placed in the forebrain along the midline where most neuronal somas are located. Electrodes were filled with 2 M NaCl and electrical activity was recorded using a Patch Clamp PC-505B amplifier (Warner Instruments, Hamden, CT). Voltage records were low-pass filtered at 2 kHz (−3 dB, 8-pole Bessel), Notch filtered at 60 Hz, digitized at 10 kHz using a Digidata 1300 A/D interface, and stored on a PC computer running Axoscope software (Molecular Devices, Sunnyvale, CA). Electrophysiological recordings were coded and analyzed post hoc using Clampfit software (Molecular Devices).

## Results

### Gene Expression

**Fig 1:**
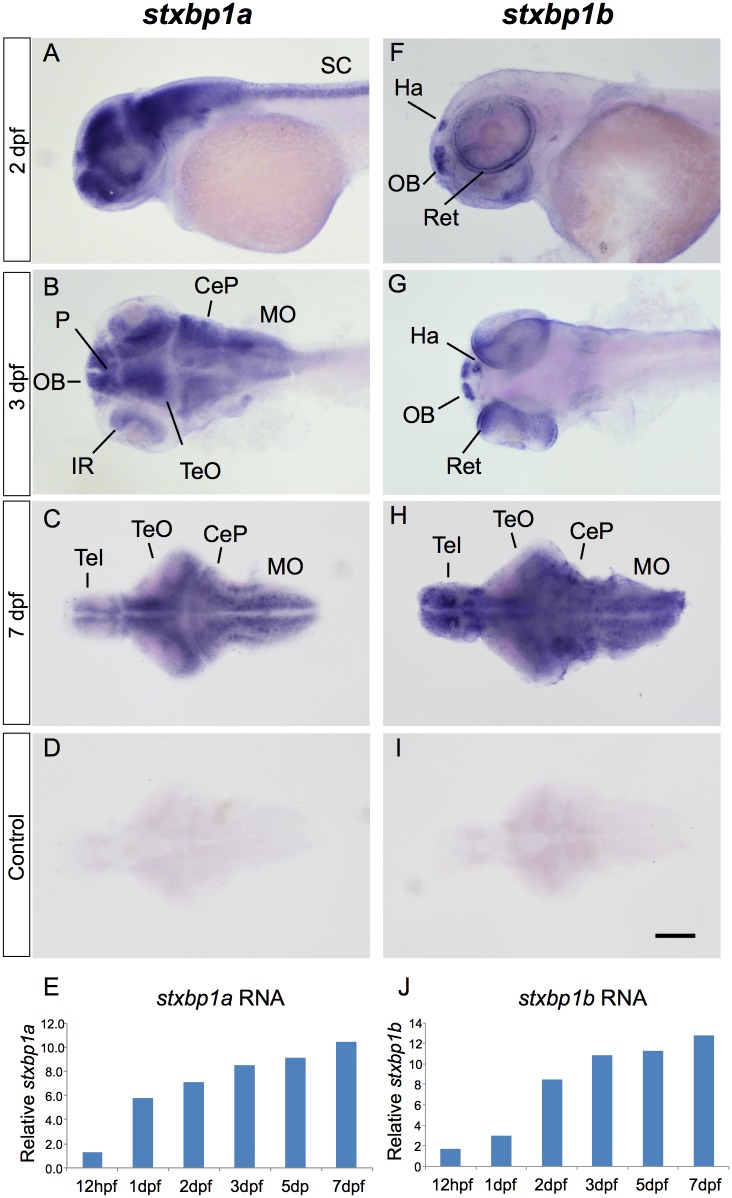
Ontogeny of *stxbp1a* and *stxbp1b* expression in zebrafish larvae. Wild-type zebrafish were probed for expression of *stxbp1a* mRNA (A-E) or *stxbp1b* mRNA (F-J). Abbreviations: CeP: cerebellar plate, Ha: habenula, IR: inner retina, MO: medulla oblongata, OB: olfactory bulb, P: pallium, Ret: retina, SC: spinal cord, Tel: telencephalon, TeO: optic tectum, Scale bar = 100μm.

We investigated the spatial expression of *stxbp1a* and *stxbp1b* in developing zebrafish using whole-mount colorimetric in situ hybridization (ISH). At 2 and 3 days post-fertilization (dpf), *stxbp1a* expression was prominent throughout the anterior-posterior axis in all major central nervous system (CNS) structures including telencephalon, optic tectum (TeO), cerebellum (CeP), medulla oblongata (MO) and spinal cord (Fig 1A and 1B). In contrast, the distribution of *stxbp1b* at 2 dpf and 3 dpf was more restricted with expression limited to the olfactory bulb (OB), the right habenula (Ha), and the outer regions of the retina (Fig 1F and 1G). In isolated 7 dpf brain, both *stxbp1a* and *stxbp1b* were also prominent in telencephalon, TeO, CeP, and MO (Fig 1C and 1H); isolated 7 dpf whole brains probed with sense control probes showed no staining (Fig 1D and 1I).

The temporal expression of *stxbp1a* and *stxbp1b* were investigated in developing zebrafish using quantitative real-time PCR. In RNA samples extracted from pooled wild-type larvae, both genes were expressed at 12 hours post-fertilization (hpf). By 1 dpf, *stxbp1a* RNA levels relative to β-actin increased 5-fold and continued a steady increase through 7 dpf (Fig 1E). In contrast, *stxbp1b* RNA levels increased less than 2-fold by 1 dpf but continued increasing rapidly until 2 dpf and beyond (Fig 1J).

### CRISPR/Cas9-Generated *stxbp1a* and *stxbp1b* Mutations

**Fig 2:**
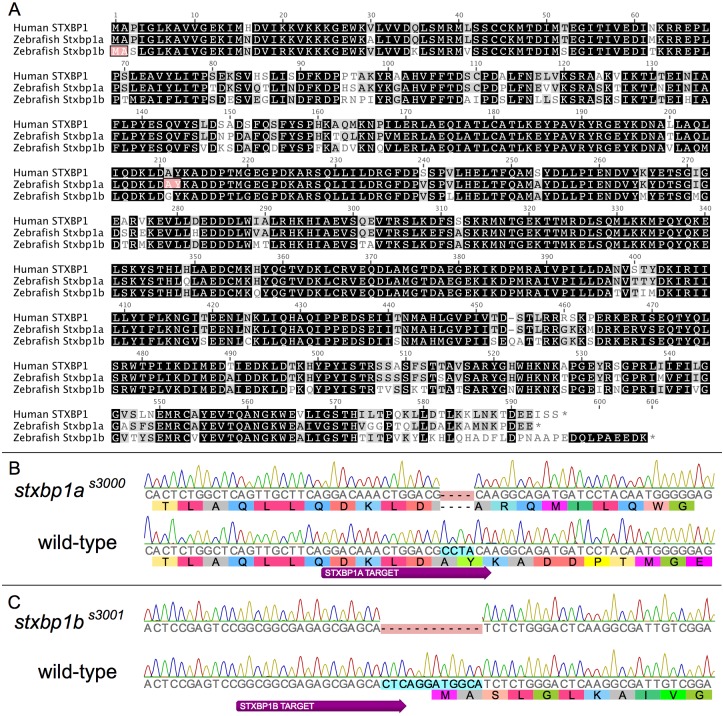
Zebrafish *stxbp1a* CRISPR/Cas9 mutant allele. (A) Sites of human and zebrafish mutations in highly conserved Stxbp1 sequence. Human STXBP1A protein sequence was aligned to zebrafish Stxbp1a and Stxbp1b. Black background indicates amino acid residues that are similar in all three proteins (BLOSUM 62). Grey background indicates amino acid residues that are similar between two of the three proteins. The salmon-colored background indicates the position of the deletion mutation in zebrafish mutant alleles. (B) Alignment of the mutant *stxbp1a*^s3000^ allele sequence (top) with wild-type zebrafish *stxbp1a* sequence (bottom). The CRISPR/Cas9 target site is shown as a wide purple arrow below the plot. The site of the 4bp deletion *stxbp1a*^s3000^ allele is highlighted in salmon, and corresponds to amino acids 211–212 highlighted in (A). (C) Alignment of the mutant *stxbp1b*^*s3001*^ allele sequence with wild-type zebrafish *stxbp1b* sequence.

The zebrafish Stxbp1a amino acid sequence (including stop codon) shares 87.2% pairwise identity with human STXBP1 sequence (Fig 2). Zebrafish Stxbp1b amino acid sequence is 78.5% identical to human STXBP1 (Fig 2). The two zebrafish paralogs, Stxbp1a and Stxbp1b, share 74.9% pairwise amino acid sequence identity.

By injecting fertilized wild-type zebrafish eggs with a mixture of Cas9 mRNA and gene-specific short guide RNA (sgRNA) we targeted germline mutations in both *stxbp1a* and *stxbp1b*. We identified F1 zebrafish (offspring of the CRISPR/Cas9-injected fish) with mutations in *stxbp1a* (Fig 2B) and outcrossed offspring of one F1 fish to generate a stable line. F2 offspring were sequenced to confirm that they shared an identical *stxbp1a* mutation. Cloning and sequencing revealed that the mutant allele is a 4 base-pair deletion (Zv9 Chromosome 21: 12,099,014–12,099,017). This deletion causes a frameshift in exon 8 of the primary *stxbp1a* transcript (Ensembl: ENSDART00000015629; GenBank: NM_001025182), and is predicted to cause a premature stop codon. This novel mutant was given the line designation *stxbp1a*^*s3000*^.

Similarly, we identified F1 zebrafish (offspring of the CRISPR/Cas9-injected fish) with mutations in *stxbp1b* (Fig 2C) and outcrossed offspring of one F1 fish to generate a stable line. F2 offspring were sequenced to confirm that they shared an identical *stxbp1b* mutation. Cloning and sequencing revealed that the mutant allele is a 12 base-pair deletion (Zv9 Chromosome 5: 30,611,750–30,611,761). This deletion causes a loss of the predicted start codon and second codon of the *stxbp1b* transcript (Ensembl: ENSDART00000005638; GenBank: NM_001089376), along with 6 upstream bases. This novel mutant was given the line designation *stxbp1b*^*s3001*^.

### Morphology

**Fig 3:**
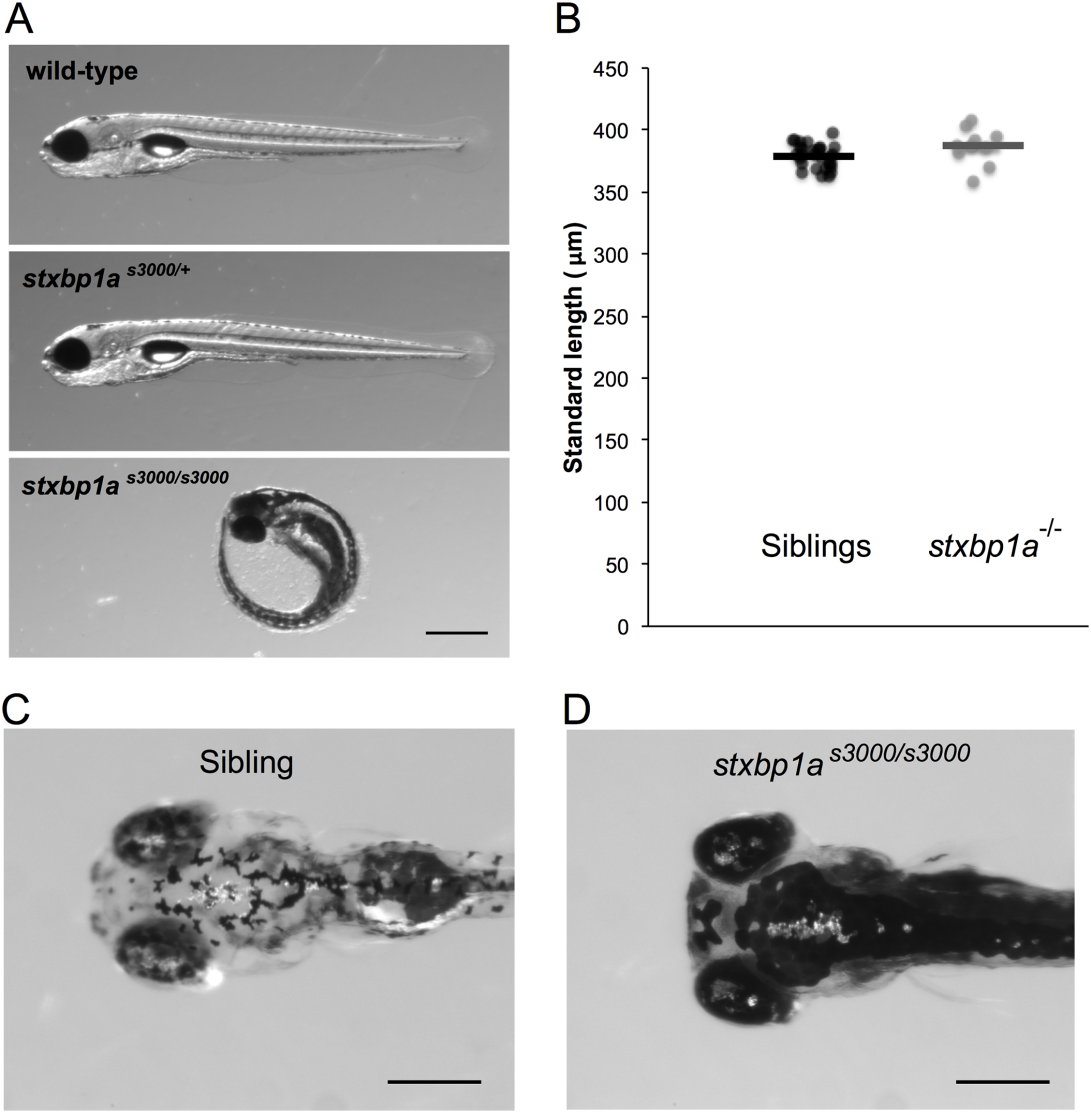
Morphology of *stxbp1a*^s3000^ mutant zebrafish. (A) Heterozygous *stxbp1a*^*s3000/+*^ mutant larvae (5 dpf) are morphologically indistinguishable from wild-type siblings. Homozygous *stxbp1a*^*s3000/s3000*^ mutant larvae are immobile and fail to hatch out of the chorion. Scale bar = 500 μm. (B) Homozygous *stxbp1a*^*s3000/s3000*^ mutant larvae (n = 10) removed from their chorions are not significantly different in length from their siblings (n = 30; p = 0.0592, two-tailed t-test). (C-D) At 5 dpf, the dorsal surface of homozygous *stxbp1a*^*s3000/s3000*^ mutant larvae that were removed from their chorions at 2 dpf (D) show dispersed melanin and foreshortened craniofacial structure compared to siblings (C). Scale bar = 300 μm.

Homozygous *stxbp1a*^*s3000/s3000*^ mutant zebrafish have abnormal morphology (Fig 3). They fail to hatch out of the chorion by 5 dpf, yielding a laterally curved body shape (Fig 3A). When they are manually released from the chorion and allowed to develop, homozygous *stxbp1a*^*s3000/s3000*^ mutant larvae (n = 10) are not significantly different in length from their control wild-type or heterozygous siblings (n = 30, p = 0.0592, two-tailed t-test; Fig 3B). The *stxbp1a*^*s3000/s3000*^ mutant larvae are also darkly pigmented, with dispersed melanin on their heads and backs. Furthermore, *stxbp1a*^*s3000/s3000*^ mutants exhibit abnormal craniofacial development, with the anterior part of the head foreshortened (Fig 3C and 3D).

**Fig 4:**
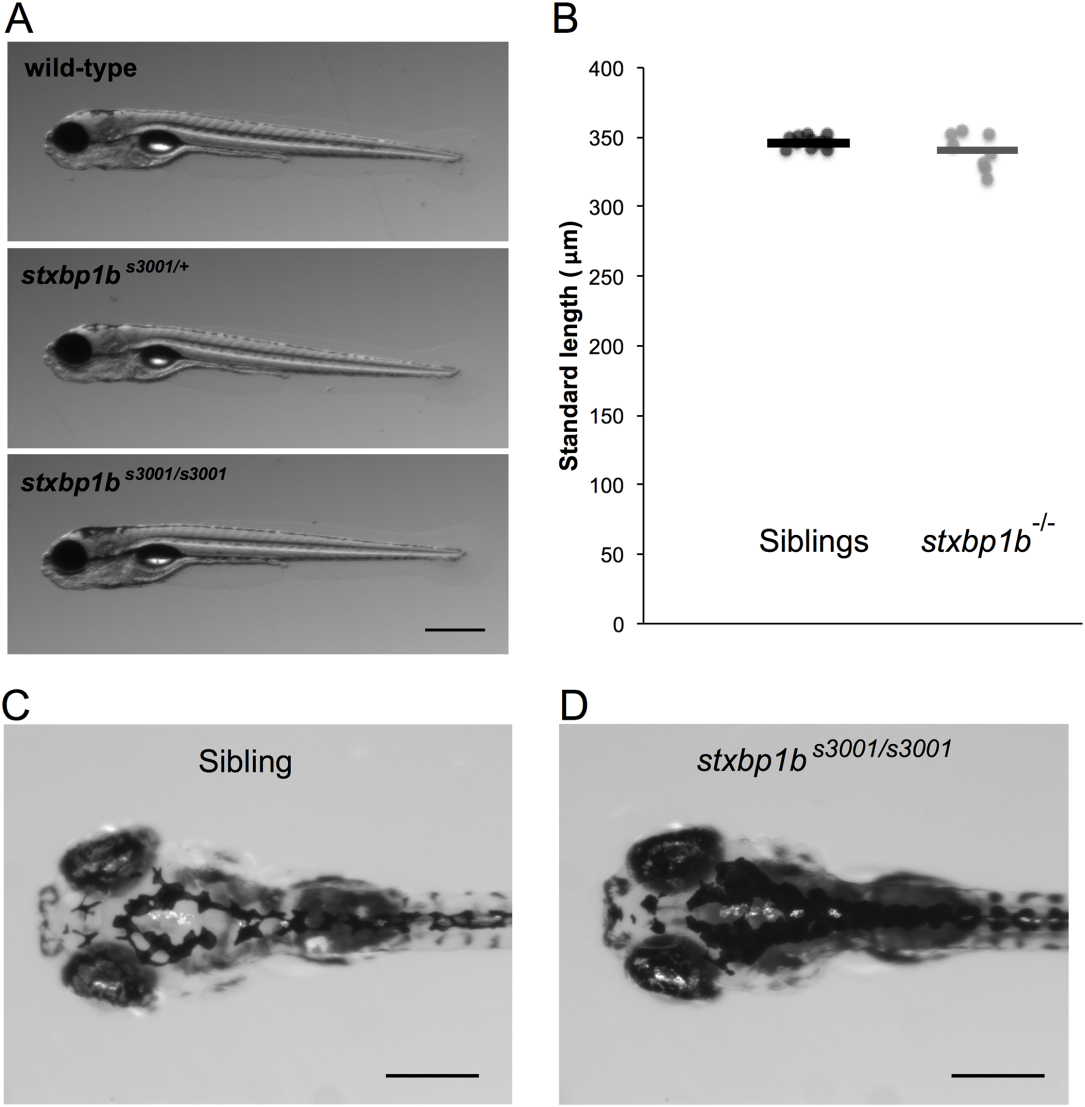
Morphology of *stxbp1b*^*s3001*^ mutant zebrafish. (A) Both heterozygous *stxbp1b*^*s3001/+*^ and homozygous *stxbp1b*^*s3001/s3001*^ mutant larvae (5 dpf) are morphologically similar to their wild-type siblings. Scale bar = 500 μm. (B) Homozygous *stxbp1b*^*s3001/s3001*^ mutant larvae (n = 8) are not significantly different in length from their siblings (n = 13) (p = 0.2297, two-tailed t-test). (C-D) The dorsal surface of homozygous *stxbp1b*^*s3001/s3001*^ mutant larvae (D) show dispersed melanin compared to siblings (C). Scale bar = 300 μm.

Conversely, homozygous *stxbp1b*^*s3001/s3001*^ mutant zebrafish have grossly normal morphology (Fig 4A). Homozygous *stxbp1b*^*s3001/s3001*^ mutant larvae (n = 10) are not significantly different in length from their control siblings (n = 30, p = 0.0592, two-tailed t-test; Fig 4B). Heterozygous mutant siblings and wild-type siblings were not significantly different from each other in length. The *stxbp1b*^*s3001/s3001*^ mutant larvae are also darkly pigmented, with dispersed melanin on their heads and backs (Fig 4C and 4D).

### Behavior

**Fig 5:**
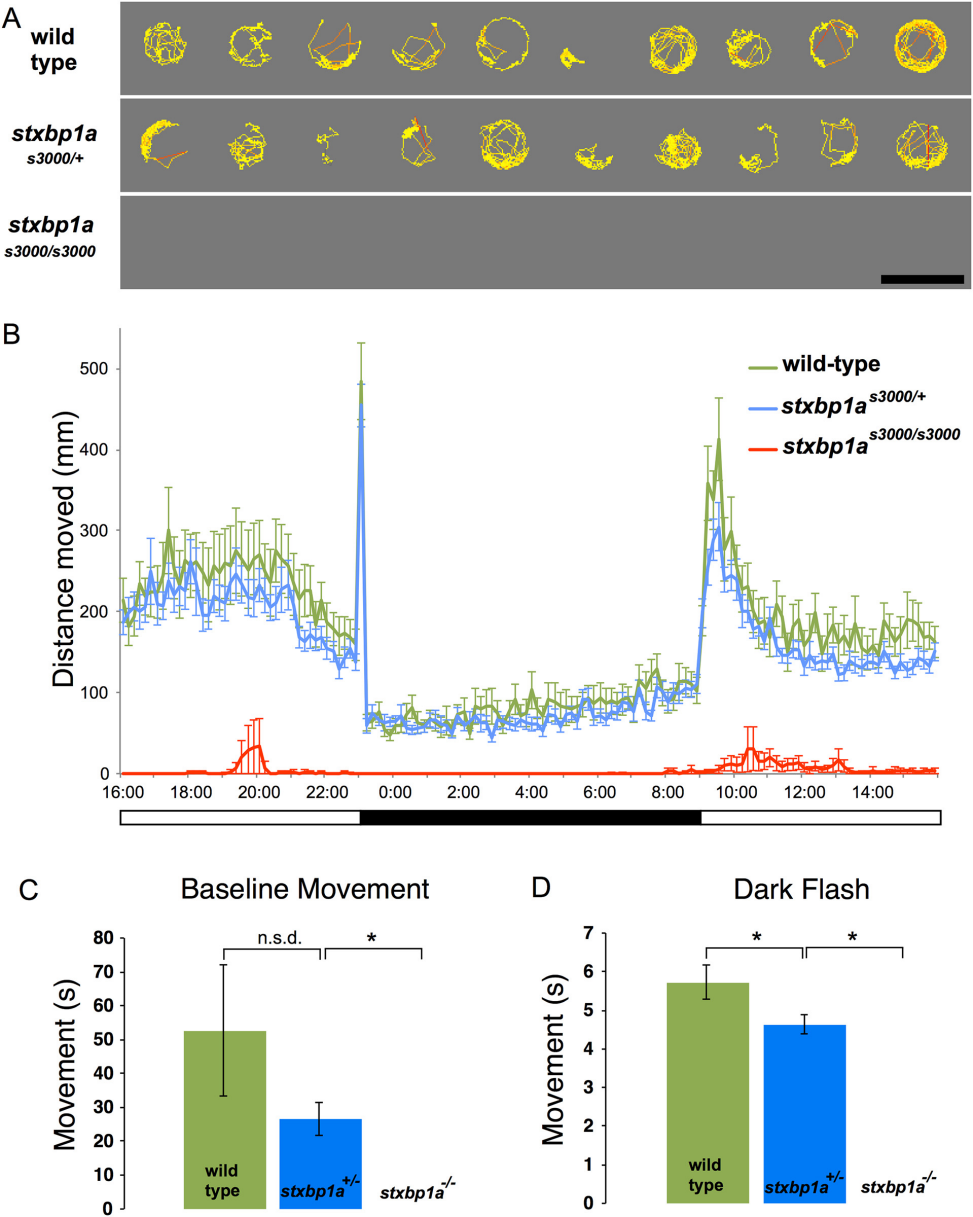
Locomotor deficits in *stxbp1a* mutant zebrafish larvae. (A) Immobility in homozygous *stxbp1a*^*-/-*^ mutant larval zebrafish. Cumulative plots of the position and velocity of 10 representative wild-type larvae, 10 representative *stxbp1a*^*s3000/+*^ heterozygous mutants, and 10 *stxbp1a*^*s3000/s3000*^ homozygous mutants during 10 minutes of behavioral recording. Larval zebrafish (5 dpf) were placed in individual wells of a flat-bottom 96-well plate and acclimated to the Daniovision recording chamber before tracking began. Yellow indicates low velocity movement; red indicates high velocity movement. No movements were detected in the homozygous mutants during this period. Scale bar = 1 cm. (B) Larval zebrafish (5 dpf) were placed in individual wells of a flat-bottom 96-well plate and acclimated to the Daniovision recording chamber. 24 hours of movement data were collected beginning at 4:00 PM. Data shown are sums of 10-minute bins ± SD (n = 23 WT, 45 Het, 21 Mut). (C) Homozygous *stxbp1a*^*s3000/s3000*^ mutants did not exhibit baseline movement (velocity = 0). There was no significant difference in movement (seconds spent moving) between heterozygous *stxbp1a*^*s3000/+*^ and wild-type siblings (shown in Fig, mean ± SD, n = 23 WT, 45 Het, 21 Mut; two-tailed t-test, p = 0.131), or for velocity or distance traveled (not shown). (D) In response to transition from 100% light intensity to darkness (0% light intensity), heterozygous larvae (6 dpf) responded with less movement than their wild-type siblings (mean ± SE, n = 23 WT, 45 Het, 21 Mut; two-tailed t-test, p = 0.042). Only one mutant responded (and moved less than 1mm).

To examine diurnal locomotor activity and responses to a known movement-inducing visual stimulus, we quantified movement of individual zebrafish larvae between 5 and 6 dpf. Neither heterozygous nor homozygous *stxbp1a* mutants displayed overt behavioral hallmarks of seizures, such as hyperactivity or whole-body convulsions (n = 23 WT, 45 Het, 21 Homo; Fig 5A). In normal embryo media, homozygous *stxbp1a*^*s3000/s3000*^ 5 dpf mutant zebrafish did not move during the initial 10-minute recording window (Fig 5A and 5C). During the subsequent 24-hour light-dark period, several homozygous mutants moved small distances (Fig 5B), but at 6 dpf none moved in response to the sudden onset of darkness for 10 seconds (a “dark flash”) (Fig 5D).

To assess the ability of *stxbp1a*^*s3000/s3000*^ mutants to respond to a seizure-inducing drug, we exposed mutants and siblings to 15 mM pentylenetetrazole (n = 12 mutants, n = 12 sibling). Sibling controls exhibit seizure behavior and a significant increase in mean swim velocity with exposure to this convulsant (baseline: 1.41 ± 0.35 mm/sec; PTZ: 3.72 ± 0.56 mm/sec; p = 0.002 two-tailed t-test); *stxbp1a*^*s3000/s3000*^ swim velocity did not change with PTZ exposure (baseline: 0 ± 0 mm/sec; PTZ: 0 ± 0 mm/sec).

Heterozygous *stxbp1a*^*s3000/+*^ 5 dpf larvae movement was not different from wild-type siblings during the initial 10-minute recording window or during most of the subsequent 24-hour light-dark period (Fig 5A–5C). In response to sudden darkness, however, heterozygous mutants exhibited significantly less movement than wild-type siblings (p = 0.042, two-tailed t-test; Fig 5D).

**Fig 6:**
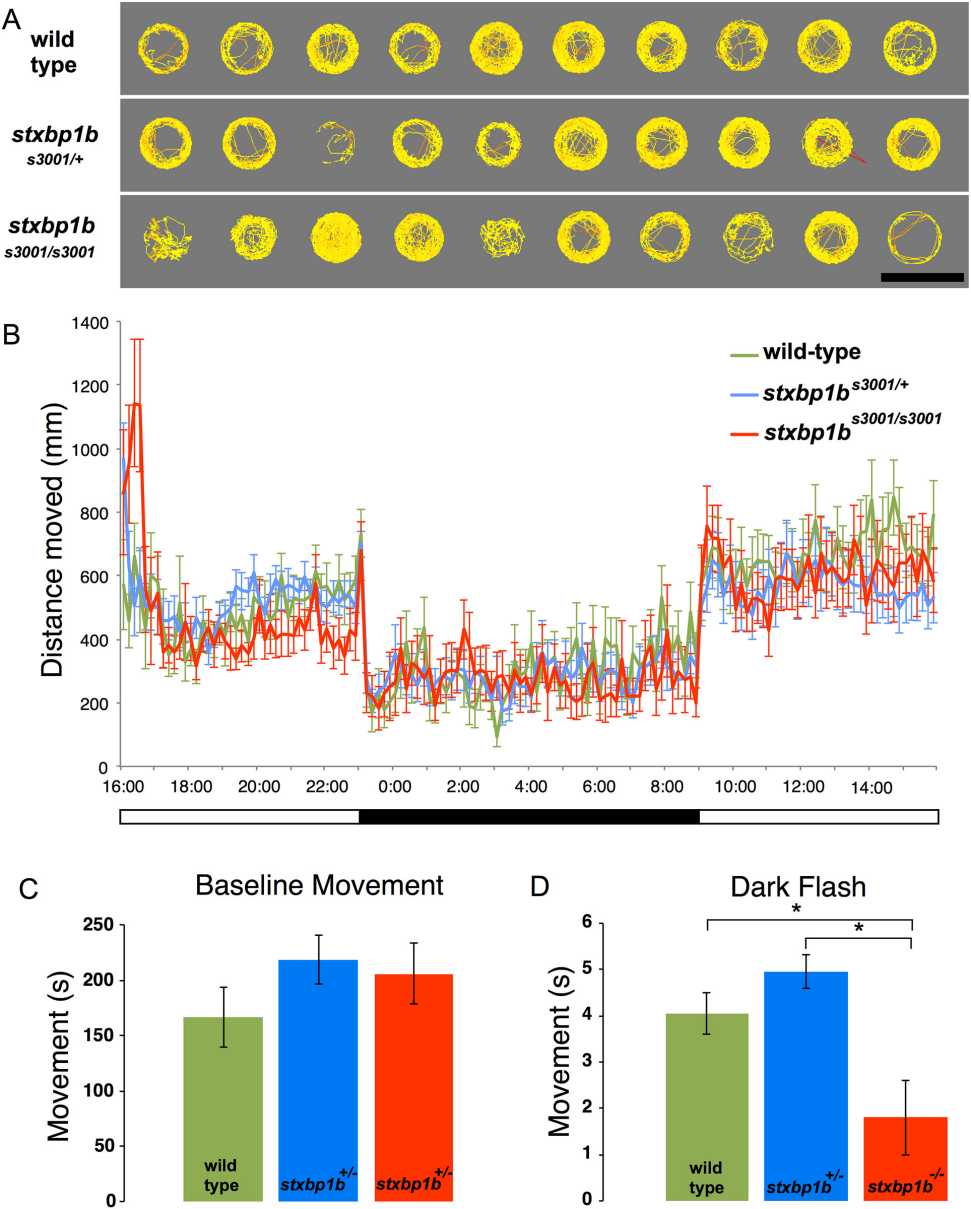
Dark-flash response deficit in *stxbp1b* mutant zebrafish larvae. (A) Normal mobility in homozygous *stxbp1b*^*-/-*^ mutant larval zebrafish. Cumulative plots of the position and velocity of 10 representative wild-type larvae, 10 representative *stxbp1b*^*s3001/+*^ heterozygous mutants, and 10 *stxbp1b*^*s30010/s3001*^ homozygous mutants during 10 minutes of behavioral recording. Larval zebrafish (5 dpf) were placed in individual wells of a flat-bottom 96-well plate and acclimated to the Daniovision recording chamber before tracking began. Yellow indicates low velocity movement; red indicates high velocity movement. Scale bar = 1 cm. (B) Larval zebrafish (5 dpf) were placed in individual wells of a flat-bottom 96-well plate and acclimated to the Daniovision recording chamber. 24 hours of movement data were collected beginning at 4:00 PM. Data shown are sums of 10-minute bins (mean ± SD; n = 13 WT, 23 Het, 12 Mut). (C) Homozygous *stxbp1b*^*s3001/s3001*^ and heterozygous *stxbp1b*^*s3001/+*^ mutants’ baseline movement did not differ statistically from wild-type baseline movement. There were no significant differences in movement (seconds spent moving) between heterozygous *stxbp1b*^*s3001/+*^ and wild-type siblings (two-tailed t-tests, n = 13 WT, 23 Het, 12 Mut), or for velocity or distance traveled (not shown; mean ± SD). (D) In response to transition from 100% light intensity to darkness (0% light intensity), homozygous *stxbp1b*^*s3001/s3001*^ larvae (6 dpf) responded with less movement than either their wild-type siblings (two-tailed t-test, p = 0.021) or their heterozygous siblings (two-tailed t-test, p = 0.00024) mean ± SE (n = 13 WT, 23 Het, 12 Mut).

Like *stxbp1a*^*s3000*^ mutants, neither heterozygous nor homozygous *stxbp1b*^*s3001*^ mutants displayed overt behavioral evidence of seizures (n = 13 WT, 23 Het, 12 Homo; Fig 6A). In normal embryo media, heterozygous *stxbp1b*^*s3001/+*^ and homozygous *stxbp1b*^*s3001/s3001*^ 5 dpf mutant zebrafish moved normally during a 10-minute recording window and subsequent 24-hour recording period (Fig 6A–6C). Homozygous *stxbp1b*^*s3001/s3001*^ did show significantly lower movement in response to sudden darkness, compared to either their wild-type siblings (two-tailed t-test, p = 0.021) or their heterozygous siblings (two-tailed t-test, p = 0.00024) (n = 13 WT, 23 Het, 12 Homo; Fig 6D).

Heterozygous *stxbp1b*^*s3001/+*^ 5 dpf larvae movement was not different from wild-type siblings during the initial 10-minute recording window, during the subsequent 24-hour light-dark period, or in response to sudden darkness (Fig 6A, 6B, 6C and 6D).

**Fig 7:**
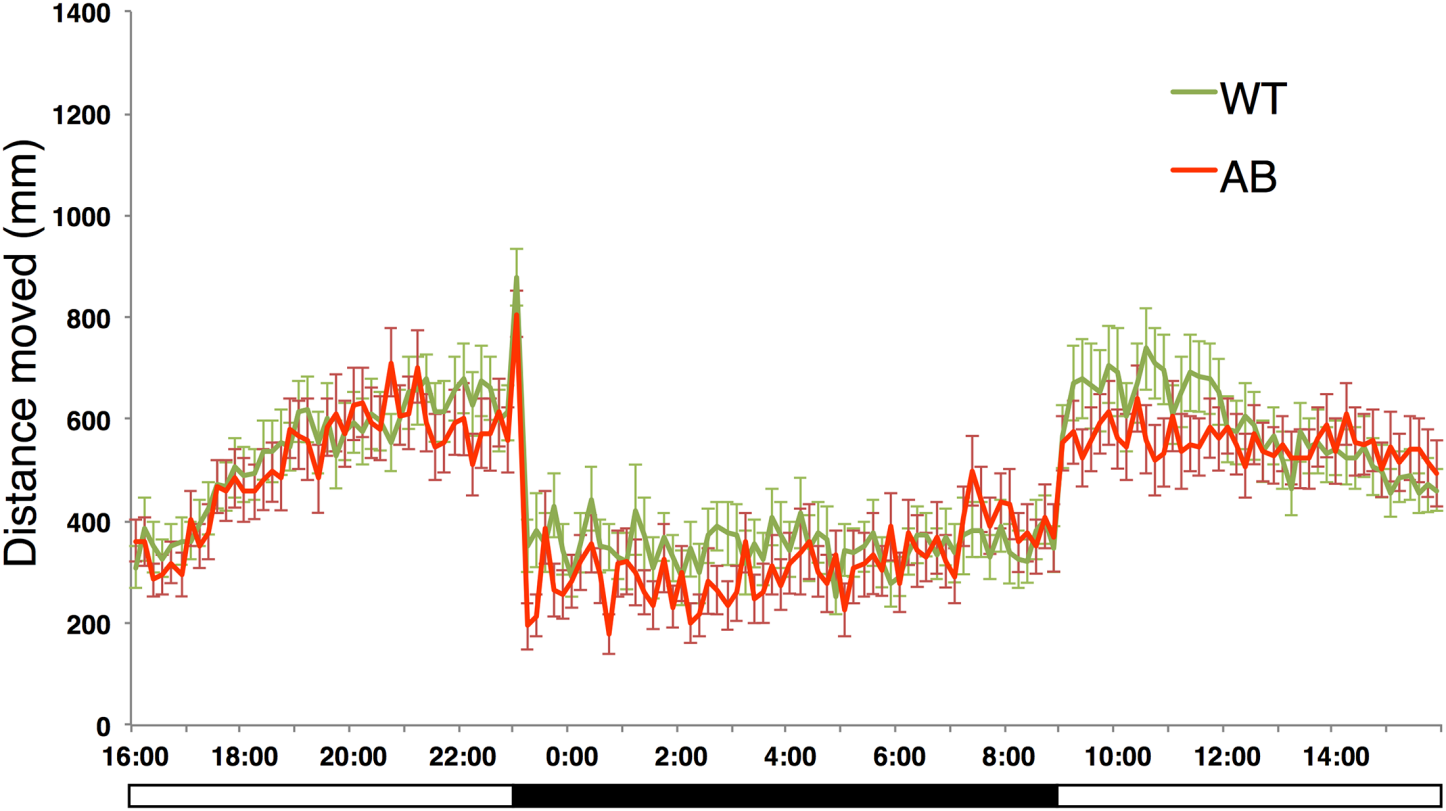
Behavior in double mutant *stxbp1a*^*s3000/+*^ *stxbp1b*^*s3001/+*^ larvae. Graph of distance traveled per ten-minute interval by 5dpf wildtype (WT, n = 16) and *stxbp1a*^*s3000/+*^ *stxbp1b*^*s3001/+*^ double mutant zebrafish (AB, n = 24) larvae over 24 hours. Mean distance per 10-min interval was 479.3 for WT larvae and 445.4 for AB larvae, significantly different by ANOVA and Tukey post hoc tests (p<0.001) as described in the main text.

We compared locomotor activity in double mutant *stxbp1a*^*s3000/+*^ *stxbp1b*^*s3001/+*^ zebrafish larvae (Fig 7). ANOVA comparison of WT (n = 27), *stxbp1a* heterozygotes (n = 16), *stxbp1b* heterozygotes (n = 24), and double *stxbp1a*/*stxbp1b* heterozygotes (n = 24) revealed a significant effect of genotype (df = 3, F = 8.460, p<0.001) and a significant effect of time (df = 143, F = 15.611, p<0.001) on distance traveled (using type III Sum of Squares). A post hoc Tukey test revealed significantly shorter distance traveled in *stxbp1a*/*stxbp1b* heterozygotes compared to WT (mean diff. = -33.834, SE = 6.866, p<0.001).

### Metabolism

**Fig 8:**
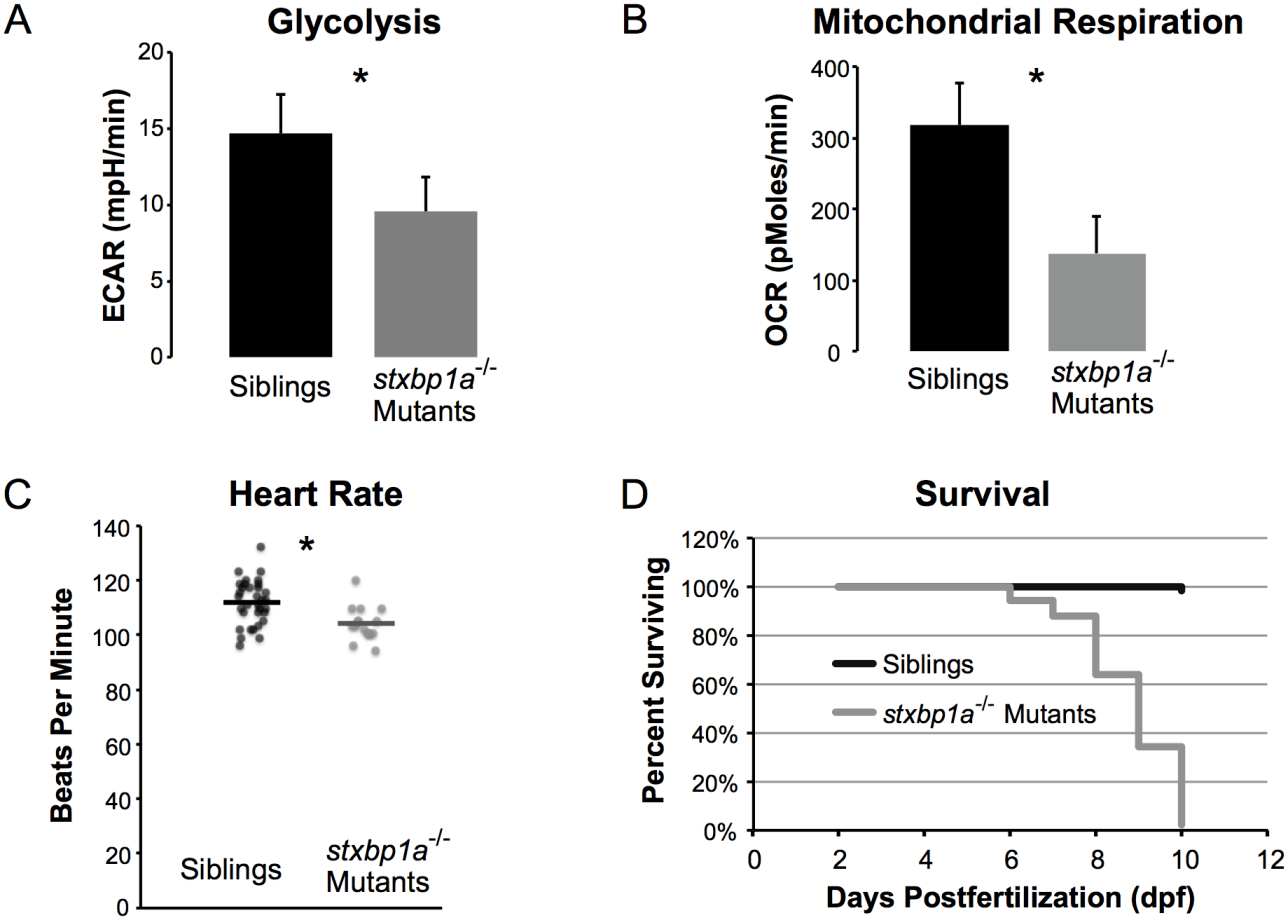
Metabolic and survival deficits caused by *stxbp1a*^s3000^ mutation. Homozygous *stxbp1a*^*s3000/s3000*^ mutant larvae have lower metabolism than controls (siblings) and die prematurely. (A) 5 dpf *stxbp1a*^*s3000/s3000*^ homozygous mutants (n = 10) had significantly lower extracellular acidification (ECAR) than siblings (n = 10; mean ± SD). * = p < 0.0001 (two-tailed t-test). (B) 5 dpf *stxbp1a*^*s3000/s3000*^ homozygous mutants (n = 10) had significantly lower oxygen consumption (OCR) than siblings (n = 10; mean ± SD). * = p < 0.0001 (two-tailed t-test). (C) Heart rates of *stxbp1a*^*s3000/s3000*^ mutant larvae (n = 15) at 3 dpf were significantly lower than heart rates of their siblings (n = 33). All measured values are plotted; mean values are indicated by horizontal lines. (D) Homozygous *stxbp1a*^*s3000/s3000*^ mutants die as larvae. Homozygous mutants (n = 50) and siblings (n = 50) were maintained in petri dishes without food and counted each day from 2 dpf until 10dpf. The *stxbp1a*^*s3000/s3000*^ mutants began dying at 6 dpf, and 98% (49) died by 10dpf. Only 2% (1) of siblings died at 10dpf.

To determine if metabolism was altered in mutant larvae, we assessed the two major energy pathways, i.e. glycolysis via extracellular acidification rates (ECAR) and mitochondrial oxidative phosphorylation via oxygen consumption rates (OCR). Baseline glycolysis rate in homozygous *stxbp1a*^*s3000/s3000*^ (n = 10) and age-matched sibling zebrafish (n = 10) were 9.54 ± 0.42 mpH/min (mean ± SD) and 14.66 ± 0.46 mpH/min respectively, indicating a 34.9% lower ECAR (p = 0.0001; Fig 8A). Similarly, OCR values were significantly lower in *stxbp1a*^*s3000/s3000*^ larvae (137.5 ± 9.53 pMoles/min) compared to age-matched siblings (317.9 ± 10.62 pMoles/min) (p < 0.0001; Fig 8D).

**Fig 9:**
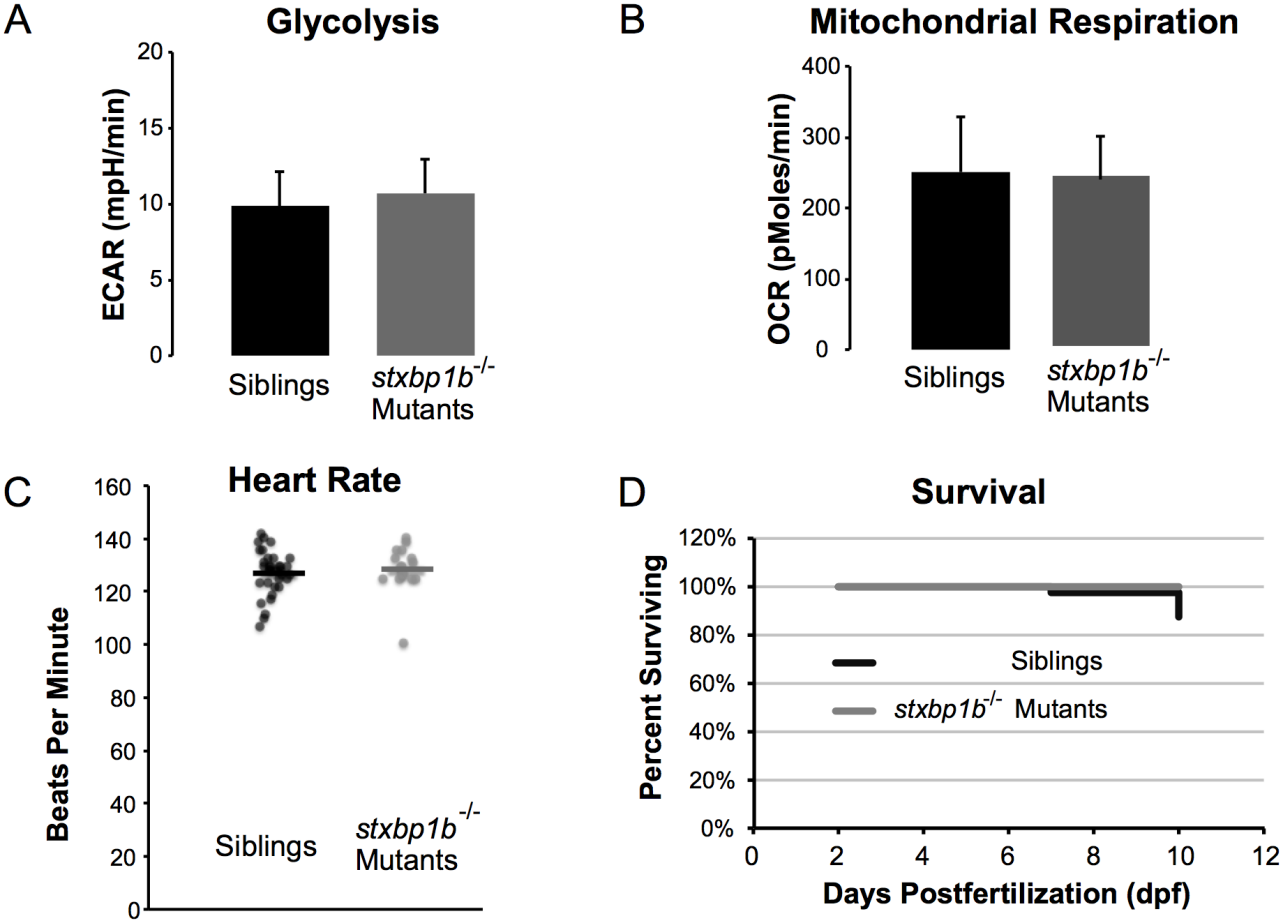
Normal metabolic function in *stxbp1b*^*s3001*^ mutants. Homozygous *stxbp1b*^*s30010/s3001*^ mutant larvae have unaffected metabolism compared to controls (siblings). (A) 5 dpf *stxbp1b*^*s30010/s3001*^ homozygous mutants (n = 11) did not differ from siblings (n = 52) in extracellular acidification (ECAR) (two-tailed t-test; mean ± SD). (B) 5 dpf *stxbp1b*^*s30010/s3001*^ homozygous mutants (n = 11) did not differ from siblings (n = 52) in oxygen consumption (OCR) (two-tailed t-test; mean ± SD). (C) Heart rates of *stxbp1b*^*s30010/s3001*^ mutant larvae (n = 16) at 3 dpf were not significantly different from heart rates of their siblings (n = 34). All measured values are plotted; mean values are indicated by horizontal lines. (D) All *stxbp1b*^*s30010/s3001*^ mutant larvae tested (n = 8) survived until 10 dpf; 12.5% of their control siblings (n = 48) died by 10dpf.

Baseline ECAR in homozygous *stxbp1b*^*s3001/s3001*^ (n = 10) and age-matched sibling zebrafish (n = 10) were not significantly different (p > 0.05; Fig 9A). Likewise, OCR was also not significantly different (p > 0.05; Fig 9B).

### Heart Rate

At 3 dpf, *stxbp1a*^*s3000/s3000*^ mutant larvae (n = 15) showed a small but statistically significant decrease in mean heart rate compared to sibling controls (n = 33; p = 0.002, two-tailed t-test; Fig 8C).

At 3 dpf, *stxbp1b*^*s3001/s3001*^ mutant larvae (n = 16) showed no difference in mean heart rate compared to sibling controls (n = 34; p = 0.550, two-tailed t-test; Fig 9C).

### Survival

Homozygous *stxbp1a*^*s3000/s3000*^ mutant larvae (n = 50) began dying at 6 dpf, four days before sibling controls (n = 50). By 10 dpf, 98% of homozygous mutants died, while only 2% of siblings died by the same age (Fig 8D).

Homozygous *stxbp1b*^*s3001/s3001*^ mutant larvae (n = 8) all survived until 10 dpf, although 12.5% of sibling controls (n = 50) died by 10 dpf (Fig 9D).

### Electrophysiology

**Fig 10:**
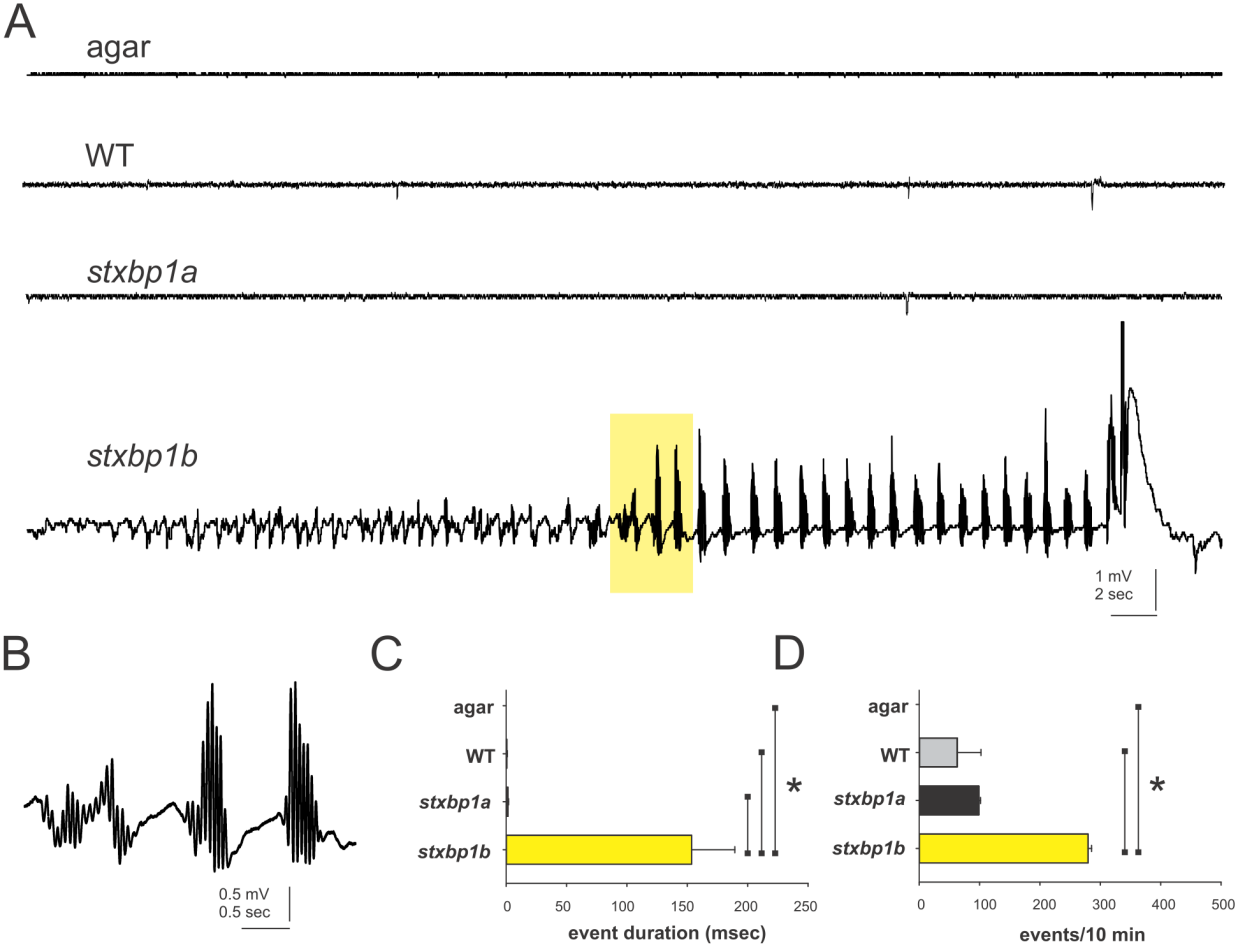
Electrophysiological phenotypes of *stxbp1a* and *stxbp1b* homozygous mutant larvae. (A) Forebrain field potential recordings from homozygous *stxbp1a*^*s3000/s3000*^ and *stxbp1b*^*s3001/s3001*^ mutant larvae compared to a control recording from an electrode in 1.2% low-melting point agarose or age-matched wild-type zebrafish; approximately 1 min of gap-free recordings are shown. (B) Magnified view of the yellow highlighted region in the above trace from a *stxbp1b*^*s3001/s3001*^ mutant larva. (C) Mean duration of all spontaneous events (greater than 50 msec in duration) was significantly greater in *stxbp1b*^*s3001/s3001*^ mutant larvae compared to *stxbp1a*^*s3000/s3000*^ larvae, WT or agar; *p < 0.001 One-way ANOVA on ranks with a Dunn’s multiple comparison test. (D) Frequency of spontaneous events was also significantly greater in *stxbp1b*^*s3001/s3001*^ mutant larvae compared to WT or agarose; *p < 0.001 One-way ANOVA on ranks with a Dunn’s multiple comparison test.

Forebrain field potential recordings from mutant larvae revealed severe and spontaneous epileptic seizure events in *stxbp1b*^*s3001/s3001*^ (n = 5) homozygous mutant larvae (Fig 10). In contrast, *stxbp1a*^*s3000/s3000*^ mutant larvae (n = 7) electrical activity was generally similar to age-matched WT controls (n = 5), with very few above-threshold events observed. Recording from the agarose mounting medium (n = 6) revealed little baseline noise.

## Discussion

Here we report the generation of *stxbp1a* and *stxbp1b* mutant zebrafish to explore the effects of *STXBP1* mutations in neurodevelopmental disorders including early infantile epileptic encephalopathy with burst suppression (EIEE). Homozygous mutation of *stxbp1a* led to severe physiological and behavioral deficits including immobility, reduced heart rate and metabolism, hyperpigmentation, and early death, while heterozygous mutation of *stxbp1a* led to generally normal behavior with only a slight reduction in response to a startling visual stimulus. Strikingly, homozygous *stxbp1b* mutants showed pronounced epileptic seizures along with mild hyperpigmentation, with normal mobility, heart rate, metabolism, and gross morphology.

### Expression

The more severe neurodevelopmental phenotypes correlated with mutation of *stxbp1a* may be related to its higher conservation and broader early CNS expression compared to *stxbp1b*. Due to an ancient whole-genome duplication in the ancestor of all teleost fishes, zebrafish have two copies of many single-copy human genes [43–46]. Following the teleost-specific genome duplication, the duplicated gene pairs (ohnologs) experienced a range of outcomes including non-functionalization, sub-functionalization, and neo-functionalization [47, 48]. Sub-functionalization may happen via protein changes [49] or via regulatory element loss [50] as described by the “duplication-degeneration-complementation” model, in which ancestral expression domains are differentially lost in different genes [51]. For example, recent evidence suggests that duplication of a broadly expressed corticotropin-releasing hormone (CRH) gene led to one onholog becoming restricted to only a sub-region of the zebrafish hypothalamus [52, 53]. On the other hand, some ohnologs retain similar expression domains and overlapping functions. For example, zebrafish have two similar homologs of the human *SCN1A* gene, *scn1laa* and *scn1lab*; both genes are expressed in the brain and homozygous loss-of-function mutation in *scn1lab* results in epilepsy and serves as a model for Dravet syndrome [34, 54, 55]. Understanding the parallel evolutionary histories of a given gene family in humans and zebrafish can facilitate the development of appropriate zebrafish models of human diseases.

In the case of *STXBP1*, early expression of *stxbp1a* and *stxbp1b* exhibit very different patterns, even though both are broadly expressed in the brain by 7 dpf. There may also be important sub-functionalization later in development due to distinct expression patterns of *stxbp1a* and *stxbp1b* at the cellular level. The restricted expression of *stxbp1b* in the olfactory bulb and habenula suggests important roles in the development of these regions or in early-developing behaviors that require the function of circuits that include these regions. Interestingly, the right dorsal habenula is activated by olfactory stimuli, suggesting that expression of *stxbp1b* in both olfactory bulb and right habenula may delineate parts of a developing neural circuit [56].

### Morphology

Like *Stxbp1* homozygous mutant mice, *stxbp1a*^*s3000/s3000*^ and *stxbp1b*^*s3001/s3001*^ larvae are normal in size [12]. The dark pigmentation of both *stxbp1a*^*s3000/s3000*^ larvae and *stxbp1b*^*s3001/s3001*^ larvae likely reflect defects in the pathway that controls pigment granule aggregation and allows adaptive matching of pigmented area to environmental light conditions. Zebrafish retina and pineal gland relay information to the hypothalamus that causes release of hormones which then control dispersion or aggregation of skin melanosomes [57–60] Dispersion of melanosomes in both *stxbp1a*^*s3000/s3000*^ larvae and *stxbp1b*^*s3001/s3001*^ larvae thus suggests that visual or neuroendocrine processes are disrupted even in the more behaviorally and metabolically normal *stxbp1b*^*s3001/s3001*^ mutants. Dispersed melanin is also seen in other mutants with impaired visual functions [61, 62], and in the epileptic *scn1lab*^s552^ homozygous mutant larvae [34, 55]. Although Stxbp1 is necessary for normal release of hormones from mouse neuroendocrine cells [63], specific neuroendocrine defects have not been documented in children harboring mutations in *STXBP1*.

During normal zebrafish postembryonic development, the head transitions from a more square shape with a ventrally located mouth to a more triangular shape with a more anterior and dorsally positioned mouth [64]. The shortened head phenotype of *stxbp1a*^*s3000/s3000*^ larvae likely reflects a problem in early neurodevelopment and may be the result of loss of neurons or a reduced progenitor pool. Another possibility is that *stxbp1a* mutation could disrupt normal development of neural crest cells, which give rise to both melanophores and craniofacial structures [65].

### Behavior

The almost complete immobility of *stxbp1a* homozygous mutant larvae is similar to the complete immobility of *Stxbp1* mutant mice [12]. We found that treatment of *stxbp1a*^*s3000/s3000*^ larvae with pentylenetetrazole (PTZ), a GABA antagonist [66], fails to induce seizures. PTZ is a potent convulsant in wild-type zebrafish, but our results suggest that it is not sufficient to generate coordinated neural activation when synaptic vesicle fusion is disrupted.

By 5 dpf, wild-type zebrafish reliably sleep during the dark phase [67, 68] and are more active during the light phase. This diurnal rhythm in behavior was severely disrupted in *stxbp1a* mutants, but not in *stxbp1b* mutants. In humans, sleep disruptions are associated with both epileptic encephalopathies (e.g. Lennox Gastaut syndrome) [69] and autism [70].

In our dark-induced movement assay, heterozygous 6 dpf *stxbp1a*^*s3000/+*^ larvae also spent significantly less time moving compared to wild-type siblings. A darkness-induced “O-bend” response is part of the normal larval zebrafish behavioral repertoire by 5 dpf [71], and can be reliably elicited by 10 s intervals of darkness [72]. Unlike the C-bend startle response induced by acoustic or mechanosensory startling stimuli, the O-bend is characterized by higher turn angle and longer latency, and does not require the Mauthner neurons [71]. The neural circuit underlying this behavioral response to a dark flash has not been characterized in detail, but is known to undergo protein-dependent habituation [73]. Heterozygous mutation of *stxbp1a*, while not significantly impairing the larvae’s ability or inclination to move in general, may disrupt their ability to respond normally to arousing stimuli. Combined with the dispersed melanosomes that we found even in light conditions in homozygous *stxbp1a* and *stxbp1b* mutants, this decreased responsiveness to dark stimuli suggests the possibility that vision may be impaired in these mutants.

### Metabolism, heart rate, and survival

Human mutations in *STXBP1* have been linked to deficits in mitochondrial respiratory chain complex IV deficits in liver [9] and impaired oxidative phosphorylation in skeletal muscle [10]. Yet cellular metabolism has not, to our knowledge, been previously investigated in animal models of *STXBP1* deficiency. The decreased mitochondrial respiration that we observed in *stxbp1a*^*s3000/s3000*^ mutant zebrafish larvae could be due directly to decreased activity of respiratory chain enzymes in neurons. Decreased glycolytic rates may be decreased to match the decreased energetic needs of the brain. Because we measured glycolysis and mitochondrial respiration at the whole-organism level, there could also be indirect effects of impaired brain development on metabolism in other tissues, for example by neuroendocrine or autonomic signaling.

As would be expected, we did not observe any gross abnormalities in heart development. We observed a small but significant decrease in mean heart rate of homozygous *stxbp1a* mutants, which could be potentially explained by several types of heart regulation, including intrinsic, neural, and hormonal effects [74, 75]. Lack of physical activity and reduced metabolism in homozygous *stxbp1a* mutants could contribute to this difference.

The early death we observed in *stxbp1a*^*s3000/s3000*^ homozygous mutant zebrafish corresponds to the known phenotype of STXBP1 homozygous mutant mice [12]. The homozygous mutant mice die shortly after birth, likely due to paralysis and failure to breathe. Larval zebrafish do not rely on circulating hemoglobin for oxygenation, as their tissues receive sufficient oxygen via diffusion and their heart rates are not sensitive to hemoglobin function [76]. Therefore, the early death phenotype we observe is unlikely to be due to insufficient tissue oxygenation. Because the *stxbp1a*^*s3000/s3000*^ homozygous mutants die before their siblings, we can exclude the possibility that they simply die from starvation. The early death phenotype can likely be explained by the increasing necessity of synaptic release for even basic autonomic survival functions during the first 10 days of life [61,62].

### Modeling human epilepsies in zebrafish

The diversity of consequences of *STXBP1* deleterious mutations seen clinically is reflected by the different phenotypes we observed in our two zebrafish mutant lines. Most remarkably, we found evidence during electrophysiology monitoring that homozygous *stxbp1b* mutant larvae are epileptic, with frequent spontaneous abnormal electrical events indicative of seizures. Our results point to *stxbp1b* mutant zebrafish as a promising model for EIEE, also known as Ohtahara syndrome [77], and represent a novel CRISPR-Cas9 generated mutant zebrafish line modeling a human form of epilepsy. The electrical phenotype we observe in *stxbp1b* mutant larvae resemble the “severe and continuous epileptic EEG abnormality” described by Ohtahara and colleagues in human patients, known as a suppression-burst pattern.

Previous efforts modeling epilepsy in zebrafish have largely relied on existing mutations generated randomly, which are not available for every gene, or on morpholino knockdown, which can generate transient effects that are often not replicable in mutant zebrafish [78]. Because we wanted to create stable and consistent loss of function, we chose instead to generate specific germline mutations in *stxbp1a* and *stxbp1b*. The mutant phenotypes that we observe likely reflect near-total loss of function of the *stxbp1a* (frameshift and premature stop codon) and *stxbp1b* (loss of start codon), respectively. Patients’ loss-of-function mutations in *STXBP1* include nonsense, missensene, frameshift, and splicing site mutations, suggesting that loss of *STXBP1* mutation is causal in EIEE [1, 4, 79]

Recent developments in CRISPR/Cas9 genome editing hold tremendous promise for replicating specific patient-derived mutations in zebrafish in this emerging era of precision medicine [80]. Given the conserved structure and function of many zebrafish homologs of human epilepsy genes, a personalized approach to editing genes in zebrafish could yield fundamental insights as well as new therapies. Furthermore, many factors influencing disease development, including genetic interaction of *STXBP1* with other loci, as well as epigenetic, developmental, and sex differences, can be mechanistically studied in model organisms including zebrafish.

## References

[1] Saitsu H, Kato M, Mizuguchi T, Hamada K, Osaka H, Tohyama J, et al De novo mutations in the gene encoding STXBP1 (MUNC18-1) cause early infantile epileptic encephalopathy. Nature genetics. 2008;40(6):782–8. Epub 2008/05/13. 10.1038/ng.150 . 18469812

[2] Tso WW, Kwong AK, Fung CW, Wong VC. Folinic acid responsive epilepsy in Ohtahara syndrome caused by STXBP1 mutation. Pediatr Neurol. 2014;50(2):177–80. Epub 2013/12/10. 10.1016/j.pediatrneurol.2013.10.006 . 24315539

[3] Otsuka M, Oguni H, Liang JS, Ikeda H, Imai K, Hirasawa K, et al STXBP1 mutations cause not only Ohtahara syndrome but also West syndrome—result of Japanese cohort study. Epilepsia. 2010;51(12):2449–52. Epub 2011/01/06. 10.1111/j.1528-1167.2010.02767.x . 21204804

[4] Allen AS, Berkovic SF, Cossette P, Delanty N, Dlugos D, Eichler EE, et al De novo mutations in epileptic encephalopathies. Nature. 2013;501(7466):217–21. Epub 2013/08/13. 10.1038/nature12439 23934111 PMC3773011

[5] Carvill GL, Weckhuysen S, McMahon JM, Hartmann C, Moller RS, Hjalgrim H, et al GABRA1 and STXBP1: novel genetic causes of Dravet syndrome. Neurology. 2014;82(14):1245–53. Epub 2014/03/14. 10.1212/WNL.0000000000000291 24623842 PMC4001207

[6] Hamdan FF, Gauthier J, Dobrzeniecka S, Lortie A, Mottron L, Vanasse M, et al Intellectual disability without epilepsy associated with STXBP1 disruption. Eur J Hum Genet. 2011;19(5):607–9. Epub 2011/03/03. 10.1038/ejhg.2010.183 21364700 PMC3083607

[7] Deprez L, Weckhuysen S, Holmgren P, Suls A, Van Dyck T, Goossens D, et al Clinical spectrum of early-onset epileptic encephalopathies associated with STXBP1 mutations. Neurology. 2010;75(13):1159–65. Epub 2010/09/30. 10.1212/WNL.0b013e3181f4d7bf . 20876469

[8] Milh M, Villeneuve N, Chouchane M, Kaminska A, Laroche C, Barthez MA, et al Epileptic and nonepileptic features in patients with early onset epileptic encephalopathy and STXBP1 mutations. Epilepsia. 2011;52(10):1828–34. Epub 2011/07/21. 10.1111/j.1528-1167.2011.03181.x . 21770924

[9] Barcia G, Barnerias C, Rio M, Siquier-Pernet K, Desguerre I, Colleaux L, et al A novel mutation in STXBP1 causing epileptic encephalopathy (late onset infantile spasms) with partial respiratory chain complex IV deficiency. Eur J Med Genet. 2013;56(12):683–5. Epub 2013/10/08. 10.1016/j.ejmg.2013.09.013 . 24095819

[10] Keogh MJ, Daud D, Pyle A, Duff J, Griffin H, He L, et al A novel de novo STXBP1 mutation is associated with mitochondrial complex I deficiency and late-onset juvenile-onset parkinsonism. Neurogenetics. 2015;16(1):65–7. Epub 2014/11/25. 10.1007/s10048-014-0431-z . 25418441 PMC6600868

[11] Harrison SD, Broadie K, van de Goor J, Rubin GM. Mutations in the Drosophila Rop gene suggest a function in general secretion and synaptic transmission. Neuron. 1994;13(3):555–66. Epub 1994/09/01. 7917291. 7917291 10.1016/0896-6273(94)90025-6

[12] Verhage M, Maia AS, Plomp JJ, Brussaard AB, Heeroma JH, Vermeer H, et al Synaptic assembly of the brain in the absence of neurotransmitter secretion. Science. 2000;287(5454):864–9. Epub 2000/02/05. . 10657302 10.1126/science.287.5454.864

[13] Weimer RM, Richmond JE, Davis WS, Hadwiger G, Nonet ML, Jorgensen EM. Defects in synaptic vesicle docking in unc-18 mutants. Nature neuroscience. 2003;6(10):1023–30. Epub 2003/09/16. 10.1038/nn1118 12973353 PMC3874415

[14] Garcia EP, Gatti E, Butler M, Burton J, De Camilli P. A rat brain Sec1 homologue related to Rop and UNC18 interacts with syntaxin. P Natl Acad Sci USA. 1994;91(6):2003–7. Epub 1994/03/15. 8134339 10.1073/pnas.91.6.2003 PMC43297

[15] Hata Y, Slaughter CA, Sudhof TC. Synaptic vesicle fusion complex contains unc-18 homologue bound to syntaxin. Nature. 1993;366(6453):347–51. Epub 1993/11/25. 10.1038/366347a0 . 8247129

[16] Pevsner J, Hsu SC, Scheller RH. n-Sec1: a neural-specific syntaxin-binding protein. P Natl Acad Sci USA. 1994;91(4):1445–9. Epub 1994/02/15. 8108429 10.1073/pnas.91.4.1445 PMC43176

[17] Dulubova I, Khvotchev M, Liu S, Huryeva I, Sudhof TC, Rizo J. Munc18-1 binds directly to the neuronal SNARE complex. P Natl Acad Sci USA. 2007;104(8):2697–702. Epub 2007/02/16. 10.1073/pnas.0611318104 17301226 PMC1815244

[18] Sudhof TC. Neurotransmitter release: the last millisecond in the life of a synaptic vesicle. Neuron. 2013;80(3):675–90. Epub 2013/11/05. 10.1016/j.neuron.2013.10.022 24183019 PMC3866025

[19] Hager T, Maroteaux G, Pont P, Julsing J, van Vliet R, Stiedl O. Munc18-1 haploinsufficiency results in enhanced anxiety-like behavior as determined by heart rate responses in mice. Behavioural Brain Research. 2014;260:44–52. Epub 2013/12/07. 10.1016/j.bbr.2013.11.033 . 24304718

[20] Laakso ML, Leinonen L, Hatonen T, Alila A, Heiskala H. Melatonin, cortisol and body temperature rhythms in Lennox-Gastaut patients with or without circadian rhythm sleep disorders. J Neurol. 1993;240(7):410–6. Epub 1993/07/01. . 8410081 10.1007/BF00867353

[21] Jansen K, Vandeput S, Van Huffel S, Lagae L. Cardiac autonomic dysfunction in West syndrome. Epilepsy Res. 2012;102(3):167–72. Epub 2012/06/26. 10.1016/j.eplepsyres.2012.05.010 . 22727657

[22] Haesemeyer M, Schier AF. The study of psychiatric disease genes and drugs in zebrafish. Current opinion in neurobiology. 2015;30:122–30. Epub 2014/12/20. 10.1016/j.conb.2014.12.002 25523356 PMC4294547

[23] McCammon JM, Sive H. Challenges in understanding psychiatric disorders and developing therapeutics: a role for zebrafish. Dis Model Mech. 2015;8(7):647–56. Epub 2015/06/21. 10.1242/dmm.019620 26092527 PMC4486859

[24] Patten SA, Armstrong GA, Lissouba A, Kabashi E, Parker JA, Drapeau P. Fishing for causes and cures of motor neuron disorders. Dis Model Mech. 2014;7(7):799–809. Epub 2014/06/29. 10.1242/dmm.015719 24973750 PMC4073270

[25] Baraban SC, Taylor MR, Castro PA, Baier H. Pentylenetetrazole induced changes in zebrafish behavior, neural activity and c-fos expression. Neuroscience. 2005;131(3):759–68. Epub 2005/02/26. S0306-4522(04)01079-6 [pii] 10.1016/j.neuroscience.2004.11.031 . 15730879

[26] Baraban SC, Dinday MT, Castro PA, Chege S, Guyenet S, Taylor MR. A large-scale mutagenesis screen to identify seizure-resistant zebrafish. Epilepsia. 2007;48(6):1151–7. Epub 2007/05/25. EPI1075 [pii] 10.1111/j.1528-1167.2007.01075.x 17521353 PMC2211740

[27] Bassuk AG, Wallace RH, Buhr A, Buller AR, Afawi Z, Shimojo M, et al A homozygous mutation in human PRICKLE1 causes an autosomal-recessive progressive myoclonus epilepsy-ataxia syndrome. American journal of human genetics. 2008;83(5):572–81. Epub 2008/11/04. 10.1016/j.ajhg.2008.10.003 18976727 PMC2668041

[28] Teng Y, Xie X, Walker S, Rempala G, Kozlowski DJ, Mumm JS, et al Knockdown of zebrafish Lgi1a results in abnormal development, brain defects and a seizure-like behavioral phenotype. Human molecular genetics. 2010;19(22):4409–20. Epub 2010/09/08. 10.1093/hmg/ddq364 20819949 PMC2957326

[29] Mahmood F, Mozere M, Zdebik AA, Stanescu HC, Tobin J, Beales PL, et al Generation and validation of a zebrafish model of EAST (epilepsy, ataxia, sensorineural deafness and tubulopathy) syndrome. Dis Model Mech. 2013;6(3):652–60. Epub 2013/03/09. 10.1242/dmm.009480 23471908 PMC3634649

[30] Arjona FJ, de Baaij JH, Schlingmann KP, Lameris AL, van Wijk E, Flik G, et al CNNM2 mutations cause impaired brain development and seizures in patients with hypomagnesemia. PLoS Genet. 2014;10(4):e1004267 Epub 2014/04/05. 10.1371/journal.pgen.1004267 24699222 PMC3974678

[31] Schubert J, Siekierska A, Langlois M, May P, Huneau C, Becker F, et al Mutations in STX1B, encoding a presynaptic protein, cause fever-associated epilepsy syndromes. Nature genetics. 2014;46(12):1327–32. Epub 2014/11/05. 10.1038/ng.3130 . 25362483

[32] Zhang X, Ling J, Barcia G, Jing L, Wu J, Barry BJ, et al Mutations in QARS, encoding glutaminyl-tRNA synthetase, cause progressive microcephaly, cerebral-cerebellar atrophy, and intractable seizures. American journal of human genetics. 2014;94(4):547–58. Epub 2014/03/25. 10.1016/j.ajhg.2014.03.003 24656866 PMC3980424

[33] Ramirez IB, Pietka G, Jones DR, Divecha N, Alia A, Baraban SC, et al Impaired neural development in a zebrafish model for Lowe syndrome. Human molecular genetics. 2012;21(8):1744–59. Epub 2012/01/03. 10.1093/hmg/ddr608 22210625 PMC3313792

[34] Baraban SC, Dinday MT, Hortopan GA. Drug screening in Scn1a zebrafish mutant identifies clemizole as a potential Dravet syndrome treatment. Nat Commun. 2013;4:2410 Epub 2013/09/05. 10.1038/ncomms3410 . 24002024 PMC3891590

[35] Westerfield M. The zebrafish book A guide for the laboratory use of zebrafish (Danio rerio). 4th ed Eugene: Univ. of Oregon Press; 2000.

[36] Livak KJ, Schmittgen TD. Analysis of relative gene expression data using real-time quantitative PCR and the 2(-Delta Delta C(T)) Method. Methods. 2001;25(4):402–8. Epub 2002/02/16. 10.1006/meth.2001.1262 . 11846609

[37] Pfaffl MW. A new mathematical model for relative quantification in real-time RT-PCR. Nucleic acids research. 2001;29(9):e45 Epub 2001/05/09. 11328886 10.1093/nar/29.9.e45 PMC55695

[38] Hwang WY, Fu Y, Reyon D, Maeder ML, Kaini P, Sander JD, et al Heritable and precise zebrafish genome editing using a CRISPR-Cas system. PLoS One. 2013;8(7):e68708 Epub 2013/07/23. 10.1371/journal.pone.0068708 23874735 PMC3706373

[39] Hwang WY, Fu YF, Reyon D, Maeder ML, Tsai SQ, Sander JD, et al Efficient genome editing in zebrafish using a CRISPR-Cas system. Nature Biotechnology. 2013;31(3):227–9. 10.1038/Nbt.2501. ISI:000316439500016. 23360964 PMC3686313

[40] Sander JD, Maeder ML, Reyon D, Voytas DF, Joung JK, Dobbs D. ZiFiT (Zinc Finger Targeter): an updated zinc finger engineering tool. Nucleic Acids Res. 2010;38(Web Server issue):W462–8. 10.1093/nar/gkq319 20435679 PMC2896148

[41] Baraban SC. Forebrain electrophysiological recording in larval zebrafish. J Vis Exp. 2013;(71). Epub 2013/02/06. 10.3791/50104 . 23380808 PMC3582514

[42] Hunt RF, Hortopan GA, Gillespie A, Baraban SC. A novel zebrafish model of hyperthermia-induced seizures reveals a role for TRPV4 channels and NMDA-type glutamate receptors. Experimental neurology. 2012;237(1):199–206. Epub 2012/06/28. 10.1016/j.expneurol.2012.06.013 22735490 PMC3418450

[43] Amores A, Catchen J, Ferrara A, Fontenot Q, Postlethwait JH. Genome evolution and meiotic maps by massively parallel DNA sequencing: spotted gar, an outgroup for the teleost genome duplication. Genetics. 2011;188(4):799–808. Epub 2011/08/11. 10.1534/genetics.111.127324 21828280 PMC3176089

[44] Christoffels A, Koh EG, Chia JM, Brenner S, Aparicio S, Venkatesh B. Fugu genome analysis provides evidence for a whole-genome duplication early during the evolution of ray-finned fishes. Mol Biol Evol. 2004;21(6):1146–51. Epub 2004/03/12. [pii]. . 15014147 10.1093/molbev/msh114

[45] Hoegg S, Brinkmann H, Taylor JS, Meyer A. Phylogenetic timing of the fish-specific genome duplication correlates with the diversification of teleost fish. J Mol Evol. 2004;59(2):190–203. Epub 2004/10/16. 10.1007/s00239-004-2613-z . 15486693

[46] Jaillon O, Aury JM, Brunet F, Petit JL, Stange-Thomann N, Mauceli E, et al Genome duplication in the teleost fish Tetraodon nigroviridis reveals the early vertebrate proto-karyotype. Nature. 2004;431(7011):946–57. Epub 2004/10/22. nature03025 [pii] 10.1038/nature03025 . 15496914

[47] Brunet FG, Roest Crollius H, Paris M, Aury JM, Gibert P, Jaillon O, et al Gene loss and evolutionary rates following whole-genome duplication in teleost fishes. Molecular biology and evolution. 2006;23(9):1808–16. Epub 2006/07/01. 10.1093/molbev/msl049 . 16809621

[48] Kassahn KS, Dang VT, Wilkins SJ, Perkins AC, Ragan MA. Evolution of gene function and regulatory control after whole-genome duplication: comparative analyses in vertebrates. Genome research. 2009;19(8):1404–18. Epub 2009/05/15. 10.1101/gr.086827.108 19439512 PMC2720184

[49] Hughes AL. The evolution of functionally novel proteins after gene duplication. Proceedings Biological sciences / The Royal Society. 1994;256(1346):119–24. Epub 1994/05/23. 10.1098/rspb.1994.0058 . 8029240

[50] Force A, Lynch M, Pickett FB, Amores A, Yan YL, Postlethwait J. Preservation of duplicate genes by complementary, degenerative mutations. Genetics. 1999;151(4):1531–45. Epub 1999/04/02. 10101175 10.1093/genetics/151.4.1531 PMC1460548

[51] Lynch M, Force A. The probability of duplicate gene preservation by subfunctionalization. Genetics. 2000;154(1):459–73. Epub 2000/01/11. 10629003 10.1093/genetics/154.1.459 PMC1460895

[52] Grone BP, Maruska KP. A second corticotropin-releasing hormone gene (CRH2) is conserved across vertebrate classes and expressed in the hindbrain of a basal Neopterygian fish, the spotted gar (Lepisosteus oculatus). The Journal of comparative neurology. 2015;523(7):1125–43. Epub 2014/12/19. 10.1002/cne.23729 . 25521515

[53] Grone BP, Maruska KP. Divergent evolution of two corticotropin-releasing hormone (CRH) genes in teleost fishes. Frontiers in Neuroscience. 2015;9 10.3389/fnins.2015.00365 PMC4602089 26528116

[54] Novak AE, Taylor AD, Pineda RH, Lasda EL, Wright MA, Ribera AB. Embryonic and larval expression of zebrafish voltage-gated sodium channel alpha-subunit genes. Developmental dynamics: an official publication of the American Association of Anatomists. 2006;235(7):1962–73. Epub 2006/04/15. 10.1002/dvdy.20811 . 16615064

[55] Schoonheim PJ, Arrenberg AB, Del Bene F, Baier H. Optogenetic localization and genetic perturbation of saccade-generating neurons in zebrafish. The Journal of neuroscience: the official journal of the Society for Neuroscience. 2010;30(20):7111–20. Epub 2010/05/21. 10.1523/JNEUROSCI.5193-09.2010 20484654 PMC3842466

[56] Dreosti E, Vendrell Llopis N, Carl M, Yaksi E, Wilson SW. Left-right asymmetry is required for the habenulae to respond to both visual and olfactory stimuli. Curr Biol. 2014;24(4):440–5. 10.1016/j.cub.2014.01.016 24508167 PMC3969106

[57] Baker BI, Ball JN. Evidence for a dual pituitary control of teleost melanophores. General and Comparative Endocrinology. 1975;25(2):147–52. Epub 1975/02/01. . 1150072 10.1016/0016-6480(75)90185-9

[58] Hogben L, Slome D. The Pigmentary Effector System. VI. The Dual Character of Endocrine Co-Ordination in Amphibian Colour Change. Proceedings of the Royal Society of London B: Biological Sciences. 1931;108(755):10–53. 10.1098/rspb.1931.0020

[59] Logan DW, Burn SF, Jackson IJ. Regulation of pigmentation in zebrafish melanophores. Pigment Cell Res. 2006;19(3):206–13. Epub 2006/05/18. 10.1111/j.1600-0749.2006.00307.x . 16704454

[60] Zhang C, Song Y, Thompson DA, Madonna MA, Millhauser GL, Toro S, et al Pineal-specific agouti protein regulates teleost background adaptation. Proc Natl Acad Sci U S A. 2010;107(47):20164–71. 10.1073/pnas.1014941107 20980662 PMC2996689

[61] Brockerhoff SE, Hurley JB, Janssen-Bienhold U, Neuhauss SC, Driever W, Dowling JE. A behavioral screen for isolating zebrafish mutants with visual system defects. Proc Natl Acad Sci U S A. 1995;92(23):10545–9. 7479837 10.1073/pnas.92.23.10545 PMC40648

[62] Neuhauss SC, Biehlmaier O, Seeliger MW, Das T, Kohler K, Harris WA, et al Genetic disorders of vision revealed by a behavioral screen of 400 essential loci in zebrafish. J Neurosci. 1999;19(19):8603–15. . 10493760 10.1523/JNEUROSCI.19-19-08603.1999 PMC6783047

[63] Voets T, Toonen RF, Brian EC, de Wit H, Moser T, Rettig J, et al Munc18-1 promotes large dense-core vesicle docking. Neuron. 2001;31(4):581–91. . 11545717 10.1016/s0896-6273(01)00391-9

[64] Parichy DM, Elizondo MR, Mills MG, Gordon TN, Engeszer RE. Normal table of postembryonic zebrafish development: staging by externally visible anatomy of the living fish. Developmental dynamics: an official publication of the American Association of Anatomists. 2009;238(12):2975–3015. Epub 2009/11/06. 10.1002/dvdy.22113 19891001 PMC3030279

[65] Eisen JS, Weston JA. Development of the neural crest in the zebrafish. Dev Biol. 1993;159(1):50–9. 10.1006/dbio.1993.1220 . 8365574

[66] Macdonald RL, Barker JL. Pentylenetetrazol and penicillin are selective antagonists of GABA-mediated post-synaptic inhibition in cultured mammalian neurones. Nature. 1977;267(5613):720–1. . 195224 10.1038/267720a0

[67] Elbaz I, Foulkes NS, Gothilf Y, Appelbaum L. Circadian clocks, rhythmic synaptic plasticity and the sleep-wake cycle in zebrafish. Front Neural Circuits. 2013;7:9 Epub 2013/02/05. 10.3389/fncir.2013.00009 23378829 PMC3561628

[68] Zhdanova IV. Sleep in zebrafish. Zebrafish. 2006;3(2):215–26. Epub 2008/02/06. 10.1089/zeb.2006.3.215 . 18248262

[69] Kerr M, Kluger G, Philip S. Evolution and management of Lennox-Gastaut syndrome through adolescence and into adulthood: are seizures always the primary issue? Epileptic Disord. 2011;13 Suppl 1:S15–26. Epub 2011/06/18. 10.1684/epd.2011.0409 . 21669559

[70] Elrod MG, Hood BS. Sleep differences among children with autism spectrum disorders and typically developing peers: a meta-analysis. J Dev Behav Pediatr. 2015;36(3):166–77. Epub 2015/03/06. . 25741949 10.1097/DBP.0000000000000140

[71] Burgess HA, Granato M. Modulation of locomotor activity in larval zebrafish during light adaptation. The Journal of experimental biology. 2007;210(Pt 14):2526–39. Epub 2007/07/03. 10.1242/jeb.003939 . 17601957

[72] Woods IG, Schoppik D, Shi VJ, Zimmerman S, Coleman HA, Greenwood J, et al Neuropeptidergic signaling partitions arousal behaviors in zebrafish. J Neurosci. 2014;34(9):3142–60. 10.1523/JNEUROSCI.3529-13.2014 24573274 PMC3935080

[73] Wolman MA, Jain RA, Liss L, Granato M. Chemical modulation of memory formation in larval zebrafish. P Natl Acad Sci USA. 2011;108(37):15468–73. Epub 2011/08/31. 10.1073/pnas.1107156108 21876167 PMC3174630

[74] Mann KD, Hoyt C, Feldman S, Blunt L, Raymond A, Page-McCaw PS. Cardiac response to startle stimuli in larval zebrafish: sympathetic and parasympathetic components. American journal of physiology Regulatory, integrative and comparative physiology. 2010;298(5):R1288–97. Epub 2010/02/05. 10.1152/ajpregu.00302.2009 . 20130228

[75] Schwerte T, Prem C, Mairosl A, Pelster B. Development of the sympatho-vagal balance in the cardiovascular system in zebrafish (Danio rerio) characterized by power spectrum and classical signal analysis. The Journal of experimental biology. 2006;209(Pt 6):1093–100. Epub 2006/03/04. 10.1242/jeb.02117 . 16513936

[76] Pelster B, Burggren WW. Disruption of hemoglobin oxygen transport does not impact oxygen-dependent physiological processes in developing embryos of zebra fish (Danio rerio). Circ Res. 1996;79(2):358–62. Epub 1996/08/01. . 8756015 10.1161/01.res.79.2.358

[77] Ohtahara S, Yamatogi Y. Ohtahara syndrome: with special reference to its developmental aspects for differentiating from early myoclonic encephalopathy. Epilepsy Res. 2006;70 Suppl 1:S58–67. 10.1016/j.eplepsyres.2005.11.021 . 16829045

[78] Kok FO, Shin M, Ni C, Gupta A, Grosse AS, van Impel A, et al Reverse Genetic Screening Reveals Poor Correlation between Morpholino-Induced and Mutant Phenotypes in Zebrafish. Dev Cell. 2014 Epub 2014/12/24. 10.1016/j.devcel.2014.11.018 . 25533206 PMC4487878

[79] Yamamoto T, Shimojima K, Yano T, Ueda Y, Takayama R, Ikeda H, et al Loss-of-function mutations of STXBP1 in patients with epileptic encephalopathy. Brain Dev. 2016;38(3):280–4. 10.1016/j.braindev.2015.09.004 . 26384463

[80] Irion U, Krauss J, Nusslein-Volhard C. Precise and efficient genome editing in zebrafish using the CRISPR/Cas9 system. Development. 2014;141(24):4827–30. Epub 2014/11/21. 10.1242/dev.115584 25411213 PMC4299274

